# Unraveling *EGFR*-TKI resistance in lung cancer with high PD-L1 or TMB in *EGFR*-sensitive mutations

**DOI:** 10.1186/s12931-023-02656-3

**Published:** 2024-01-18

**Authors:** Wuwu Ding, Pengmin Yang, Xiaokai Zhao, Xiaozhi Wang, Huaqing Liu, Qing Su, Xintao Wang, Jieyi Li, Ziying Gong, Daoyun Zhang, Xinwei Wang

**Affiliations:** 1 Department of Pathology, Deyang Pelple’s Hospital, No.173 Taishan Road, Jingyang District, Deyang City, Sichuan Province 618300 China; 2 Department of Oncology, Jiangsu Cancer Hospital & Jiangsu Institute of Cancer Research & Affiliated Cancer Hospital of Nanjing Medical University, No.42 Baiziting, Xuanwu District, Nanjing, 210009 China; 3 Jiaxing Key Laboratory of Precision Medicine and Companion Diagnostics, Jiaxing Yunying Medical Inspection Co., Ltd, Jiaxing, 314000 China; 4 Department of R&D, Zhejiang Yunying Medical Technology Co., Ltd., Building 5, 3556 Linggongtang Road, Nanhu District, Jiaxing, Zhejiang 314000 China

**Keywords:** EGFR-TKIs, PD-L1, TMB, Resistance, PI3K signaling pathway

## Abstract

**Background:**

Although EGFR-TKI resistance mechanisms in non-small cell lung cancer (NSCLC) have been extensively studied, certain patient subgroups remain with unclear mechanisms. This retrospective study analysed mutation data of NSCLC patients with *EGFR*-sensitive mutations and high programmed death-ligand 1 (PD-L1) expression or high TMB to identify primary resistance mechanisms.

**Methods:**

Hybrid capture-based next-generation sequencing (NGS) was used to analyse mutations in 639 genes in tumor tissues and blood samples from 339 NSCLC patients. PD-L1 immunohistochemical staining was also performed on the same cell blocks. Molecular and pathway profiles were compared among patient subgroups.

**Results:**

TMB was significantly higher in lung cancer patients with *EGFR*-sensitive mutations and high PD-L1 expression. Compared with the high-expression PD-L1 or high TMB and low-expression or TMB groups, the top 10 genes exhibited differences in both gene types and mutation rates. Pathway analysis revealed a significant mutations of the PI3K signaling pathway in the *EGFR*-sensitive mutation group with high PD-L1 expression (38% versus 12%, p < 0.001) and high TMB group (31% versus 13%, p < 0.05). Notably, *PIK3CA* and *PTEN* mutations emerged as the most important differentially mutated genes within the PI3K signaling pathway.

**Conclusions:**

Our findings reveal that the presence of PI3K signaling pathway mutations may be responsible for inducing primary resistance to EGFR-TKIs in NSCLC patients with *EGFR*-sensitive mutations along with high PD-L1 expression or high TMB. This finding is of great significance in guiding subsequent precision treatments in NSCLC.

**Supplementary Information:**

The online version contains supplementary material available at 10.1186/s12931-023-02656-3.

## Background

Approximately 20% of individuals diagnosed with non-small cell lung cancer (NSCLC) harbor a distinct genetic mutation in the epidermal growth factor receptor (*EGFR*) gene, referred to as an activating somatic mutation [1]. The most frequently observed mutations within this category include the exon 19 deletion (E19del) and the exon 21 substitution at position 858 (L858R), in which the amino acid arginine is substituted with leucine [2, 3]. Small molecule tyrosine kinase inhibitors (TKIs) targeting *EGFR*, including first/second-generation TKIs (gefitinib, erlotinib, and afatanib) and third-generation TKIs (osimertinib), have been extensively employed in the treatment of NSCLC patients with *EGFR*-sensitive mutations and have exhibited favorable outcomes [4–7]. However, there was significant variability in response duration and survival among these patients.

Previous studies have revealed a spectrum of resistance mechanisms to *EGFR* inhibitors, including *EGFR*-dependent resistance, such as the C797X mutation, which confers resistance to osimertinib [8], and non-*EGFR*-dependent resistance caused by the activation of bypass or downstream signaling pathways [9, 10], as well as histological or phenotypic transformation [11]. Additionally, the resistance mechanisms remain unidentified in some patients. Studies have indicated that NSCLC patients with *EGFR* mutations (E19del/L858R) accompanied by a higher tumor mutational burden (TMB) tend to have a less favorable prognosis when treated with *EGFR*-TKIs than those with low TMB [12]. Furthermore, another study has suggested that in NSCLC patients with *EGFR* mutations (E19del/L858R) and high programmed death-ligand 1 (PD-L1) expression, the prognosis with *EGFR*-TKI treatment is less favorable than that with low PD-L1 expression [13]. Nevertheless, the underlying mechanisms responsible for the poorer prognosis in these subgroups of *EGFR* mutations have not yet been thoroughly investigated.

To explore the potential mechanisms underlying these subgroups, in this study, we conducted a retrospective analysis of the genetic mutation data of patients in these NSCLC subgroups and analysed the differences in pathway mutations. Our research aimed to offer new treatment opportunities for patients with these specific types of NSCLC.

## Materials and methods

### Patients and sample characteristics

From November 2022 to August 2023, a total of 339 patients with pathologically diagnosed NSCLC who had not received *EGFR*-TKI treatment were enrolled in the present study at the Affiliated Cancer Hospital of Nanjing Medical University and Deyang Pelple’s Hospital. Each patient underwent a pathological diagnosis and was needed to provide both tumor tissue and paired blood samples. Cancer diagnosis was initially established through clinical and X-ray findings and later confirmed via histological analysis of tumor biopsies. Exclusion criteria for the study included cases where NSCLC was not pathologically confirmed, cases where tissue or blood samples were not provided, and cases where the cell blocks of the samples contained tumor cells in quantities less than 20%. Clinical data, including information on age and gender, were retrieved from the medical records. Written informed consent was obtained from all participants, and this study was approved by the institutional review board of our hospital.

### DNA extraction and library construction

According to the manufacturer’s protocols, tumor DNA and blood genomic DNA were extracted using a human tissue DNA extraction kit (Shanghai YunYing) and a human blood genomic DNA extraction kit (Shanghai YunYing), respectively. DNA was eluted in an elution buffer, and its concentration and purity were evaluated using a NanoDrop spectrophotometer. DNA was stored at -20 °C until use. Library preparation was performed using the VAHTS Universal DNA Library Prep Kit for Illumina. Target enrichment was performed using Shanghai YunYing’s optimized probes, which target the exons and some introns of 639 cancer-related genes. Sequencing was performed on an Illumina NextSeq500 platform using the manufacturer’s protocols.

### Next-generation sequencing (NGS)-based assay and bioinformatics analysis

FastQC software (version 0.11.2) and customized Python script were used to screen FASTQ files, with the adaptor sequences and sequences with Q below 30 removed. Clean reads were mapped to the reference human genome GRCh37/hg19 using BWA (Burrows Wheeler Aligner version 0.7.7). BAM files were then realigned and recalled using GATK3.5, which was also used to detect mutations. Duplicate sequences were removed using Picard MarkDuplicates (version 1.35) to reduce any potential polymerase chain reaction bias. VarScan (version 2.3.2) was used to select single nucleotide variations (SNVs) satisfying the following criteria: depth ≥ 100, reads ≥ 10, and allele frequency ≥ 5% (if hotspot, ≥ 1%). Pindel (version 0.2.5b8) was used for insertion or deletion (indel) detection using default parameters, with at least 5 unique reads.

Compared with matched normal samples, somatic SNVs and InDels of tumors were named and functionally annotated using MuTect v. 1.1.4 and Varscan2 v. 2.3.9 software. Mutations with a variant allele frequency of ≥ 5% were defined as high-confidence mutations (≥ 1% for hotspots). Tumor mutational burden (TMB) was calculated using the number of all somatic, coding, base substitution, and indel mutations per megabase including synonymous mutations. The total number of mutations counted was divided by the size of the coding region of the targeted territory (1.36 Mb of the coding genome) to calculate the TMB per megabase. Microsatellite instability (MSI) scores of all samples were calculated using MSIsensor [14] with default parameters, a software tool for quantifying MSI in genome sequencing data using tumor-only or paired tumor-normal samples. We used 29 microsatellite sites as input files for MSI detection of tumor-only patterns. The MSI score was defined as the percentage of unstable microsatellites among all microsatellites used. Each microsatellite site had at least 20 spanning reads and single-nucleotide mutations.

### PD-L1 expression test

The PD-L1 expression level for each patient was determined using the Dako 22C3 pharmDx system (Agilent Technologies Inc., Santa Clara, CA, USA) assay, and the results are presented as a tumor proportion score (TPS) [15].

### Statistical analysis

The prevalence and distribution of genomic alterations were visualized using the R package “maftools” [16]. The R package “ggplot2” was used to draw the boxplots. The nonparametric Wilcox test was subsequently used to test for the significance of the difference in means between the two populations.

## Results

### Patient characteristics

A total of 339 patients, consisting of 295 with lung adenocarcinoma (LUAD) and 44 with lung squamous cell carcinoma (LUSC), who were newly diagnosed with NSCLC cancer, were included in the present study. The mean age at diagnosis of the study participants was 61.9 years (range, 25–86 years; median, 64 years), and a significant difference was observed between PD-L1 expression level groups (Table 1). Additional characteristics of the patient cohort are summarized in Table 1, and more detailed information can be found in Table S1. All participants successfully completed the targeted sequencing, which included all exons and partial introns of the 639 genes listed in Table S2.

**Table 1:** Association between PD-L1 expression status and clinical features

Characteristics	All (N = 339)	PD-L1 expression level			^a^P-value
		High (N = 74)	Medium (N = 79)	Negative (N = 186)	
**Pathological type**, ***n (%)***					0.398
Adenocarcinoma	295 (87.0%)	62 (83.8%)	67 (84.8%)	166 (89.2%)	
Squamous cell carcinoma	44 (13.0%)	12 (16.2%)	12 (15.2%)	20 (10.8%)	
**Gender**, ***n (%)***					0.069
Female	169 (49.9%)	32 (43.2%)	48 (60.8%)	89 (47.8%)	
Male	170 (50.1%)	42 (56.8%)	31 (39.2%)	97 (52.2%)	
**Age at diagnosis in years**, ***n (%)***					0.026
60- (< 60)	131 (38.6%)	33 (44.6%)	38 (48.1%)	60 (32.3%)	
60+ (≥ 60)	208 (61.4%)	41 (55.4%)	41 (51.9%)	126 (67.7%)	
***EGFR*status**, ***n (%)***					0.138
E19del	66 (19.5%)	12 (16.2%)	18 (22.8%)	36 (19.4%)	
L858R	102 (30.1%)	14 (18.9%)	25 (31.6%)	63 (33.9%)	
other	27 (8.0%)	8 (10.8%)	4 (5.1%)	16 (8.6%)	
Wild type	144 (42.5%)	40 (54.1%)	32 (40.5%)	71 (38.2%)	
**TMB status**, ***n (%)***					0.003
TMB-H (≥ 4.4 muts/Mb)	110 (32.4%)	36 (48.6%)	24 (30.4%)	50 (26.9%)	
TMB-L (< 4.4 muts/Mb)	229 (67.6%)	38 (51.4%)	55 (69.6%)	136 (73.1%)	
**MSI status**, ***n (%)***					0.117
MSI-H	3 (0.9%)	1 (1.4%)	2 (2.5%)	0 (0.0%)	
MSS	336 (99.1%)	73 (98.6%)	77 (97.5%)	186 (100.0%)	

^a^P value are tested by chi-square test

### TMB exhibits significant differences among various clinical indicators and molecular features

**Fig. 1:**
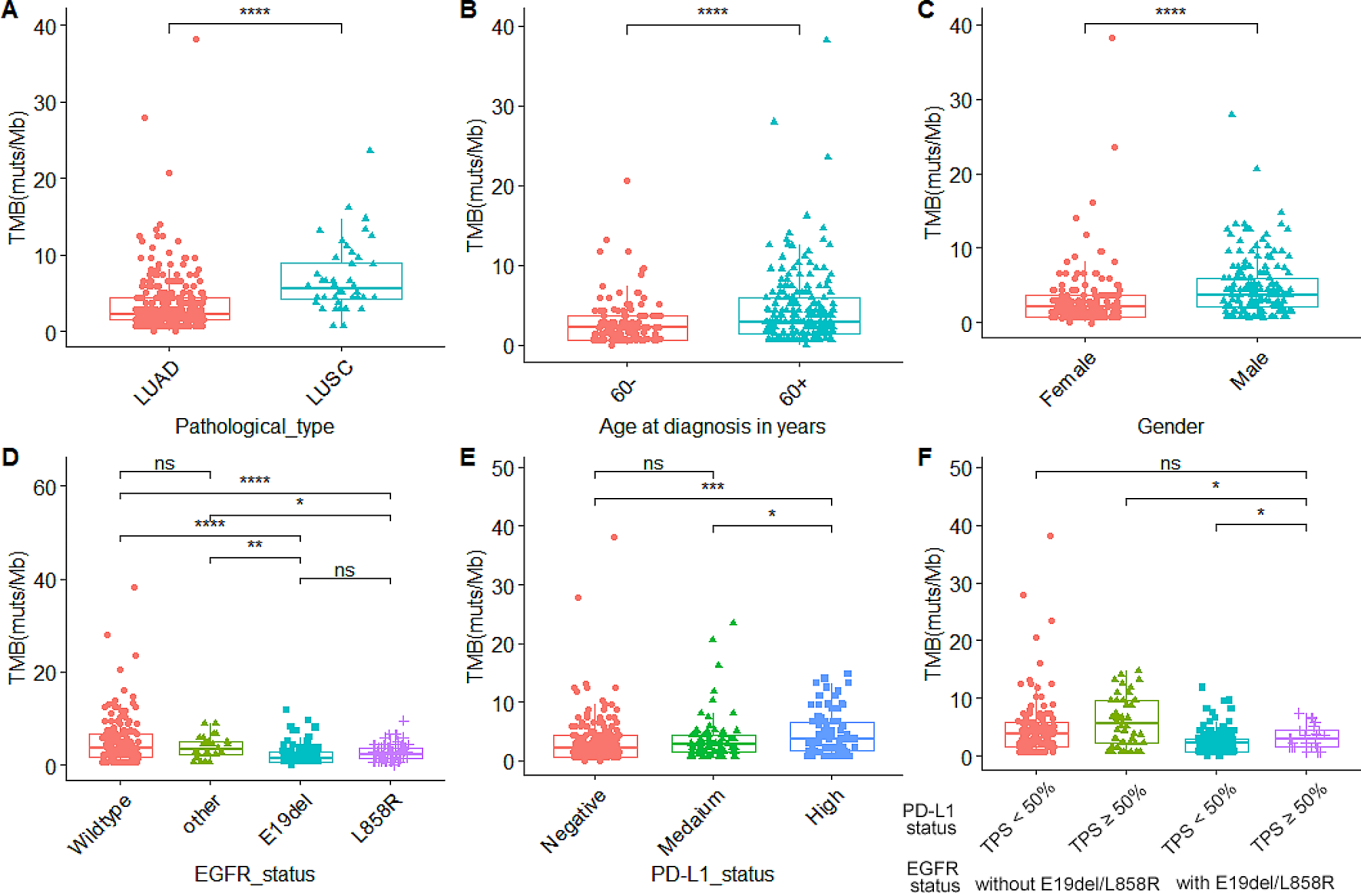
The relationship between TMB and clinical indicators. (**A**) Lung cancer pathological type; (**B**) Age at diagnosis in years; (**C**) Gender; (**D**) *EGFR* mutation status; (**E**) PD-L1 expression level; and (**F**) *EGFR* mutation status and PD-L1 expression level. “ns”, “*”, “**”, “***” and “****” indicate P > 0.05, P < 0.05, P < 0.01, P < 0.001 and P < 0.0001, Wilcoxon test. LUAD for adenocarcinoma; LUSC for squamous cell carcinoma

As shown in Fig. 1A, LUAD exhibited significantly lower TMB values than LUSC (average: 3.4 muts/Mb versus 6.8 muts/Mb, p < 0.0001). Patients aged > 60 years had higher TMB values than those aged < 60 years old (average: 4.5 muts/Mb versus 2.8 muts/Mb, p < 0.0001) (Fig. 1B). In terms of gender differences, males display significantly higher TMB values than females (average: 4.7 muts/Mb versus 3.0 muts/Mb, p < 0.0001) (Fig. 1C). Regarding molecular features, no significant differences in TMB values were observed between *EGFR* E19del and L858R mutations. Similarly, no notable distinctions were found between the other *EGFR* mutations and the wild-type (as shown in Fig. 1D). However, when looking at specific *EGFR* mutations, TMB values were notably lower for *EGFR* E19del (average: 2.5 muts/Mb versus 3.8 muts/Mb, p < 0.01; 2.5 muts/Mb versus 5.3 muts/Mb, p < 0.0001) or L858R (average: 2.6 muts/Mb versus 3.8 muts/Mb, p < 0.05; 2.6 muts/Mb versus 5.3 muts/Mb, p < 0.0001) in comparison to other *EGFR* mutations or wild-type. Likewise, no significant differences were observed in TMB between the PD-L1 negative and PD-L1 moderate expression groups. However, the high PD-L1 expression group exhibited significantly higher TMB values when compared to the PD-L1 negative or moderate expression groups (average: 4.9 muts/Mb versus 3.4 muts/Mb, p < 0.001; 4.9 muts/Mb versus 3.6 muts/Mb, p < 0.05) (Fig. 1E).

According to the results of Fig. 1D and E, we divided the patients into PD-L1 high or nonhigh-expression groups (TPS ≥ 50% or TPS < 50%), *EGFR*-sensitive or non-*EGFR*-sensitive groups (with or without *EGFR* E19del/L858 mutation), and TMB high (TMB-H) or low (TMB-L) groups (TMB ≥ 4.4 muts/Mb or TMB < 4.4 muts/Mb, greater than or less than the third quartile). High PD-L1 expression was associated with a significantly higher TMB than nonhigh PD-L1 expression (average: 3.2 muts/Mb versus 2.4 muts/Mb, p < 0.05) in patients harboring EGFR-sensitive mutations (Fig. 1F). According to the data analysis, MSI did not affect the TMB value (Fig. S1). More information is displayed in Table S3.

### Mutation overview and analysis with PD-L1 status

Among the observed mutation types, missense mutations were the most common, followed by frameshift deletions and nonsense mutations (see Fig. S2A). In terms of variant types, single nucleotide polymorphisms (SNPs) constituted a larger portion than insertions or deletions (as illustrated in Fig. S2B). Notably, the C > T transition was the dominant single nucleotide variant (SNV) observed in NSCLCs (Fig. S2C). The number of altered bases in each sample and a summary of the variant classifications were counted, as depicted in Fig. S2D and Fig. S2E, respectively. In NSCLCs, the top 10 mutated genes were *EGFR* (57%), *TP53* (46%), *LRP1B* (10%), *PIK3CA* (8%), *KRAS* (8%), *FAT1* (6%), *KEAP1* (6%), *ATM* (5%), *CDKN2A* (5%), and *NF1* (4%) (Fig. S2F) which might play an important role in the biological processes of NSCLC. According to the waterfall plot of the top 20 mutated genes, where the mutation type is denoted by various colors with annotations, nonsense and missense mutations were mostly observed (Fig. S2G).

**Fig. 2:**
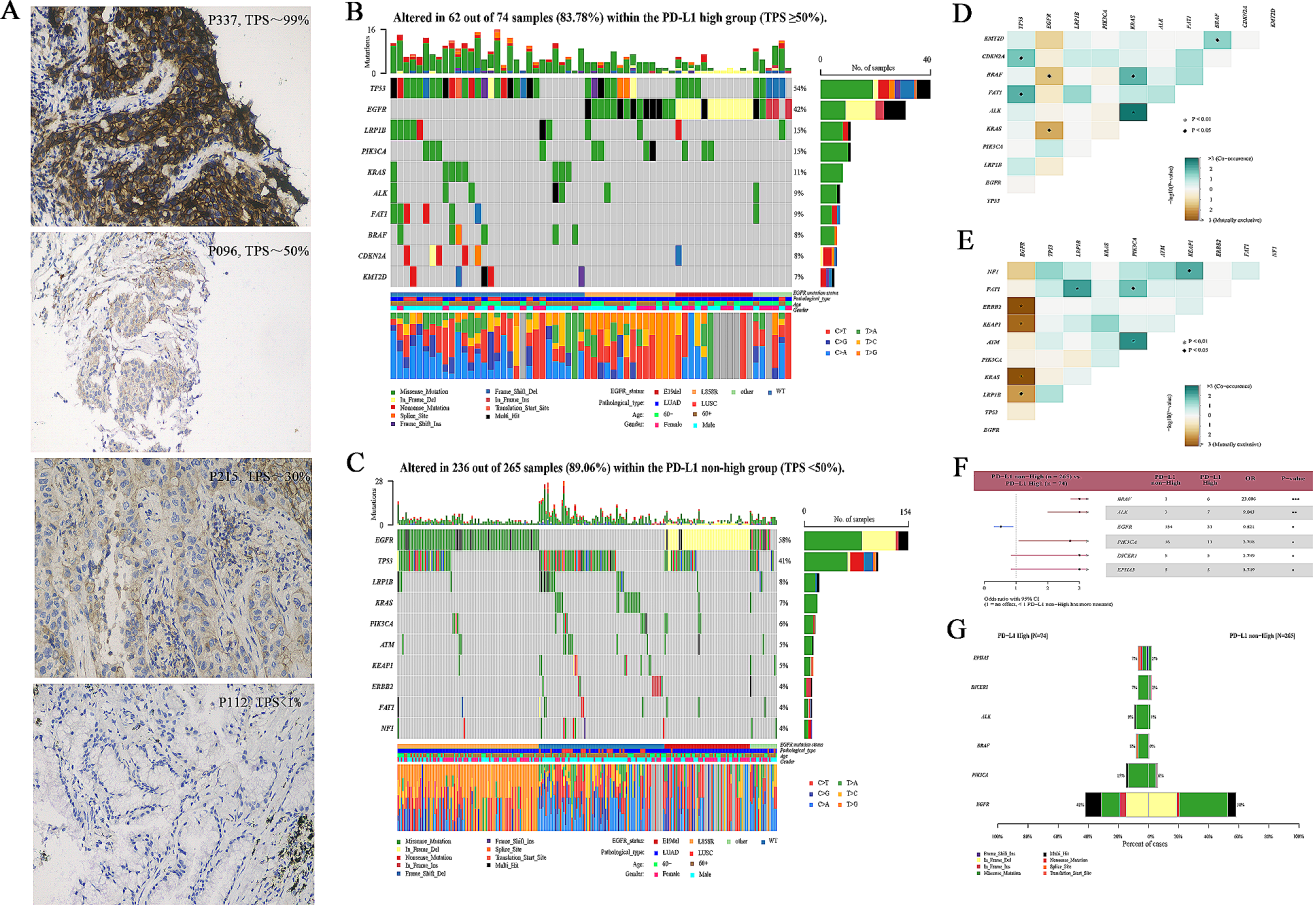
Mutation analysis in the high and nonhigh PD-L1 expression groups. (**A**) Immunohistochemical image of lung cancer patients with high expression of PD-L1 (TPS ≥ 50%) and nonhigh expression (TPS < 50%); (**B**) Overview of mutation profiles in patients with high expression of PD-L1; (**C**) Overview of mutation profiles in patients with nonhigh expression of PD-L1; (**D**) Comutation analysis in patients with high expression of PD-L1; (**E**) Comutation analysis in patients with nonhigh expression of PD-L1; (**F**) Forest plot of comparing mutations between patients with high expression and nonhigh expression of PD-L1; (**G**) Co-bar plot of differentially mutated genes in patients with high expression and nonhigh expression of PD-L1. “*”, “**”, and “***” indicate P < 0.05, P < 0.01, and P < 0.001

Figure 2 A presents the PD-L1 staining results of four representative NSCLC patients, corresponding to high and low expression levels. To understand the mutation difference in distinct PD-L1 expression groups, we plotted the mutation profiles of the top 10 mutated genes. As depicted in Fig. 2B, the top 10 genes in the PD-L1 high-expression group were *TP53, EGFR, LRP1B, PK3CA, KRAS, ALK FAT1, BRAF, CDKN2A*, and *KMT2D.* Conversely, in the nonhigh-expression group, the top 10 genes were *EGFR, TP53, LRP1B, KRAS, PK3CA, ATM, KEAP1, ERBB2, FAT1*, and *NF1* (Fig. 2C). These profiles also revealed differences in the gene mutation rates between the two groups. In the high PD-L1 expression group, several significant associations and mutual exclusions among the gene mutations were observed. Specifically, *TP53* mutations were significantly associated with *CDKN2A* mutations, and *KRAS* mutations were significantly associated with *FAT1* mutations. Conversely, *BRAF* and *ALK* mutations were mutually exclusive with *EGFR* mutations and mutually exclusive with *BRAF* and *KRAS* mutations (Fig. 2D). In the nonhigh PD-L1 expression group, comutated genes included *FAT1*, *LRP1B*, and *PIK3CA*, as well as *NF1* and *KEAP1*, and *ATM* and *PIK3CA*. *EGFR* mutations were significantly mutually exclusive with *ERBB2*, *KRAS*, *KEAP1*, and *LRP1B* mutations (see Fig. 2E). These associations and exclusions shed light on the complex genetic relationships among different PD-L1 expression groups.

Through a comparative analysis of mutations between the high and nonhigh PD-L1 expression groups, we identified significant differences in the mutation frequency of several genes: *BRAF* (8% versus 0%), *ALK* (9% versus 1%), *EGFR* (42% versus 58%), *PIK3CA* (15% versus 6%), *DICER1* (7% versus 2%) and *EPHA5* (7% versus 2%) (Fig. 2F and G) (P < 0.05).

### Mutation analysis with TMB status

**Fig. 3:**
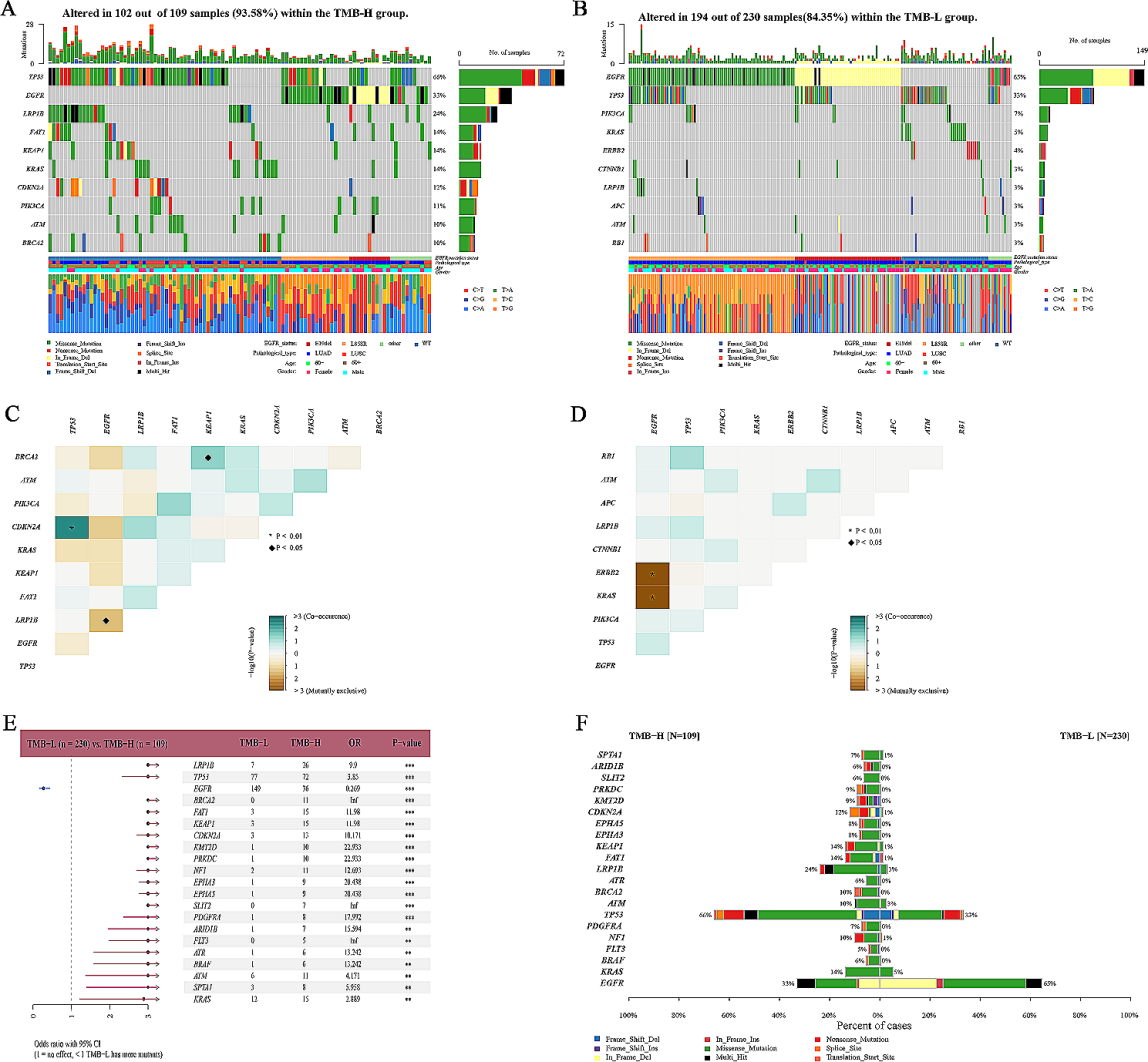
Mutation analysis in groups with high or low TMB values. (**A**) Overview of mutation profiles in patients with high tumor mutational burden (TMB-H, TMB ≥ 4.4 muts/Mb); (**B**) Overview of mutation profiles in patients with low tumor mutational burden (TMB-L, TMB < 4.4 muts/Mb); (**C**) Comutation analysis in patients with high tumor mutational burden; (**D**) Comutation analysis in patients with low tumor mutational burden; (**E**) Phylogenetic tree comparing mutations between patients with high and low tumor mutational burden; (**F**) Cobar plot of differentially mutated genes in patients with high and low tumor mutational burden. “**” and “***” indicate P < 0.01 and P < 0.001

Similarly, we generated mutation profiles for the top 10 mutated genes based on TMB status. In the TMB-H group, the top 10 genes were *TP53, EGFR, LRP1B, FAT1, KEAP1, KRAS, CDK2NA, PIK3CA, ATM*, and *BRCA2*, whereas in the low TMB group, they were *EGFR, TP53, PK3CA, KRAS, ERBB2, CTNNB1, LRP1B, APC, ATM* and *RB1*, and there were also differences in gene mutation rates (Fig. 3A and B). In the TMB-H group, *TP53* mutation and *KEAP1* were significantly associated with *CDKN2A* mutation and *BRCA2* mutations, respectively (Fig. 3C). *EGFR* mutations were significantly mutually exclusive to *LRP1B.* In the TMB-L group, *EGFR* mutations were significantly mutually exclusive to *ERBB2* and *KRAS* (Fig. 3D). Comparative mutation analysis of TMB-H and TMB-L groups showed that multiple genes are related to TMB: *SPIA1* (7% versus 1%), *ARID1B* (6% versus 0%), *SLIT2* (6% versus 0%), *PRKDC* (9% versus 0%), *KMT2D* (9% versus 0%), *CDKN2A* (12% versus 1%), *EPHA5* (8% versus 0%), *EPHA3* (8% versus 0%), *KEAP1* (14% versus 1%), *FAT1* (14% versus 1%), *LRPIB* (24% versus 3%), *ATR* (6% versus 0%), *BRCA2* (10% versus 0%), *ATM* (10% versus 3%), *TP53* (66% versus 33%), *PDGFRA* (7% versus 0%), *NF1* (10% versus 1%), *FLT3* (5% versus 0%), *BRAF* (6% versus 0%), *KRAS* (14% versus 5%), *and EGFR* (33% versus 55%)(Fig. 3E and F) (P < 0.01).

### Tumor signaling pathway analysis

To further investigate the potential impact of high PD-L1 expression or high TMB on the tumor signaling pathways in NSCLC patients with *EGFR* mutations, we conducted a tumor signaling pathway mutation analysis (pathways and related genes referenced in Table S2 in the previous study [17]). As shown in Table S4, among the *EGFR* mutated group with PD-L1 expression, there were differences in the mutation rates of the following pathways: chromatin, histone modifiers, genome integrity, histone modification, mitogen-activated protein kinase (MAPK) signaling, other signaling, PI3K (phosphoInositide 3-kinase) signaling, RNA abundance, receptor tyrosine kinase (RTK) signaling, splicing and target of rapamycin (TOR) signaling. Similarly, among the *EGFR* mutated group with TMB (Table S5), there were differences in the mutation rates of the following pathways: cell cycle, chromatin histone modifiers, chromatin SWI/SNF (SWItch/Sucrose Non-Fermentable) complex, genome integrity, MAPK signaling, other, other signaling, PI3K signaling, RTK signaling, transforming growth factor beta (TGFB) signaling, and transcription factor.

**Fig. 4:**
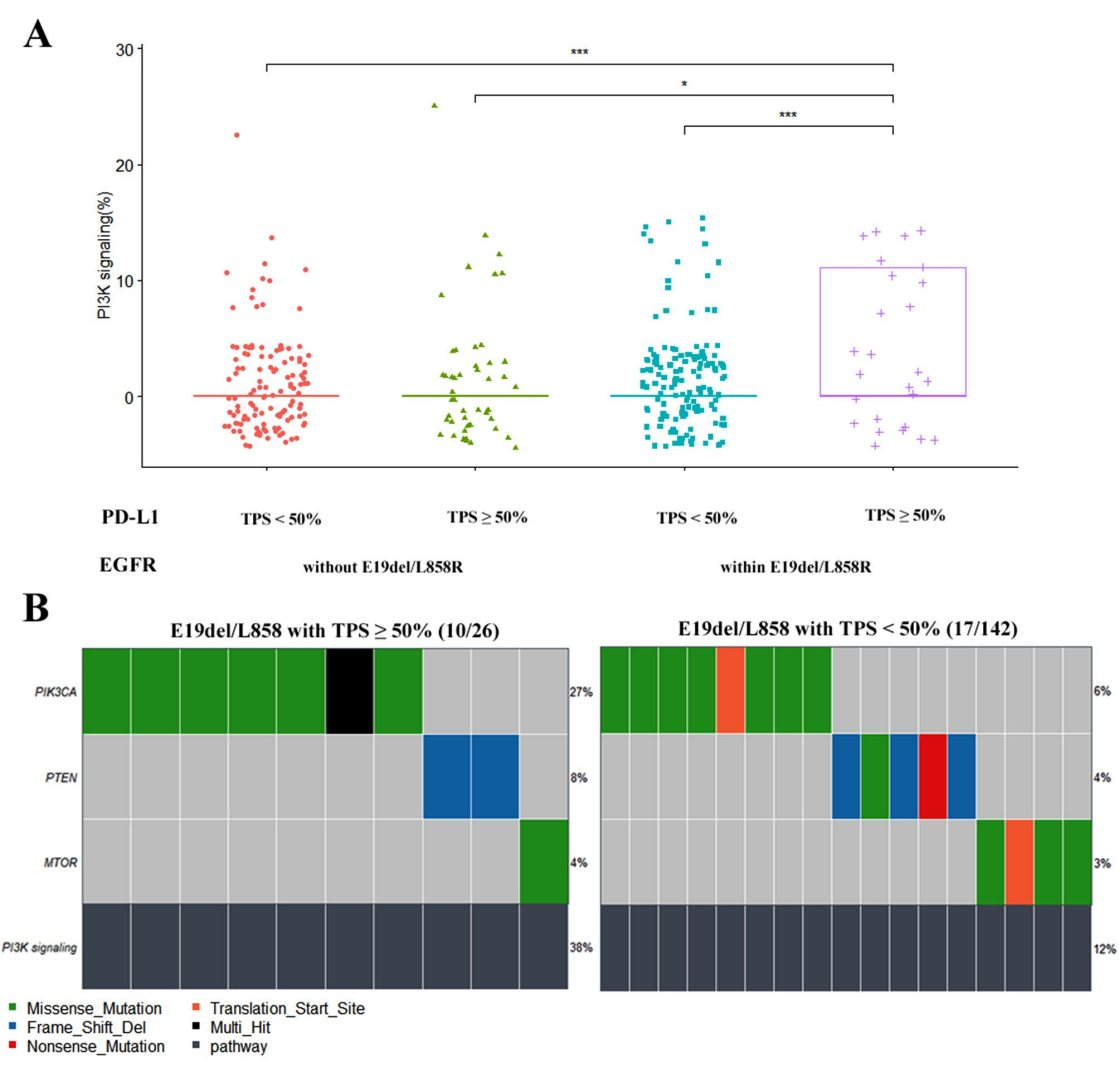
Pathway mutation differential and profile analysis in groups with high or nonhigh PD-L1 expression. Differential analysis of the PI3K signaling pathway (**A**); The mutation profile of the PI3K signaling pathway in the group with *EGFR* E19del/L858R mutation and high ( TPS ≥ 50%) or nonhigh (TPS < 50%) PD-L1 expression levels (**B**). “*” and “***” indicate P < 0.05 and P < 0.001, respectively. Wilcox test

**Fig. 5:**
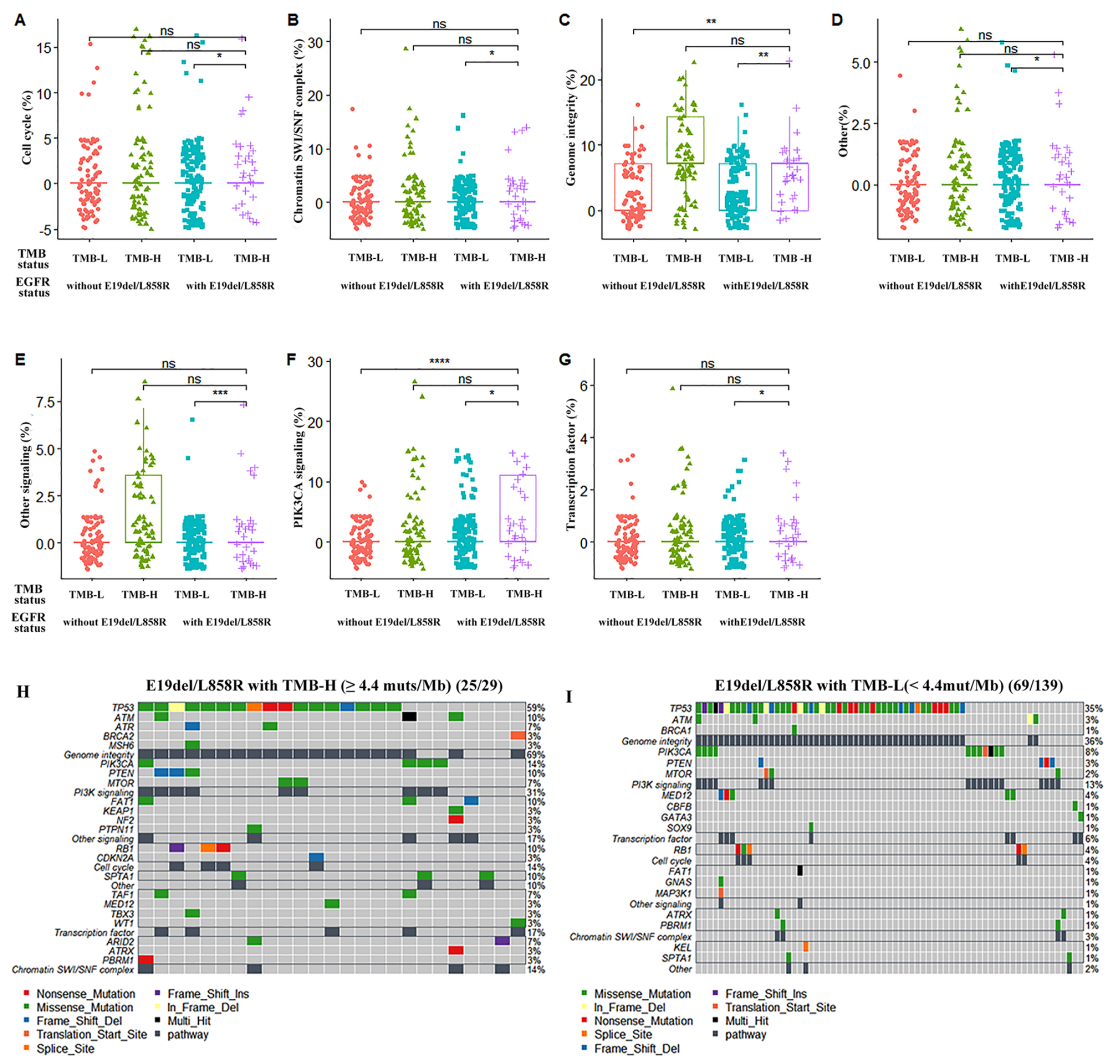
Pathway mutation differential and profile analysis in the high or low TMB value group. Differential analysis of signaling pathways: cell cycle (**A**), chromatin SWI/SNF complex (**B**), genome integrity (**C**), other (**D**), other signaling (**E**), PI3K signaling (**F**), and transcription factor (**G**); the mutation profile of signaling pathways in the group with *EGFR* E19del/L858R and high (H, TMB ≥ 4.4 muts/Mb) or low (I, TMB < 4.4 muts/Mb) TMB values. “ns”, “*”, “**”, “***” and “****” indicate P > 0.05, P < 0.05, P < 0.01, P < 0.001 and P < 0.0001, Wilcoxon test

These pathways were selected for further differential analysis. In the context of *EGFR*-sensitive mutations, accompanied by high PD-L1 expression compared to low expression, we identified a substantial difference in the mutation rates of the PI3K signaling pathway (38% versus 12%, p < 0.001) pathways (Fig. 4). In *EGFR*-sensitive mutations accompanied by TMB-H compared to TMB-L, there were significant differences in the mutation rates of the cell cycle (14% versus 3%, p < 0.05), chromatin SWI/SNF (14% versus 4%, p < 0.05), Genome. integrity (69% versus 36%, p < 0.01), Other (10% versus 2%, p < 0.01), Other signaling (17% versus 2%, p < 0.001), PI3K signaling (31% versus 13%, p < 0.05), and Transcription factor (17% versus 6%, p < 0.05) pathways (Fig. 5). The analysis of mutation differences in other selected pathways between groups is displayed in Figures S3 and S4, and no significant difference was observed in the group of *EGFR*-sensitive mutations with high versus low PD-L1 expression or high versus low TMB values. Specific mutations of the key genes *PIK3CA* and *PTEN* in the PI3K pathway are shown in the lollipop plot (Figure S5). *PIK3CA* primarily has activating mutations such as H1407, E545, or E542 (Figures S5A and S5C), while *PTEN* mainly has loss-of-function mutations (Figures S5B and S5D).

## Discussion

*EGFR*-TKI is a crucial therapy for NSCLC patients with *EGFR* mutations, but there are significant variations in their prognosis ^4–7^. Prior research has highlighted factors such as primary and acquired [18–20], tumor histology, and phenotype transformation [11] as potential reasons for these differences. Notably, recent studies have indicated that high TMB or high PD-L1 expression plays a significant role as a primary resistance mechanism to *EGFR*-TKIs in *EGFR-mutated* NSCLC. However, the specific resistance mechanisms of these distinct *EGFR* mutations remain poorly understood. In our study, we further categorized patients with NSCLC based on their PD-L1 expression levels and TMB values, in addition to their EGFR mutation status. We conducted a detailed analysis of mutation characteristics within these subgroups.

To the best of our knowledge, our study represents the first evidence suggesting that NSCLC patients with *EGFR*-sensitive mutations, coupled with high PD-L1 expression, exhibit elevated TMB. While PD-L1 and TMB are typically regarded as two distinct immune markers, our findings suggest a potential correlation between PD-L1 and TMB in the context of *EGFR*-sensitive mutations. TMB is generally defined as the number of somatic mutations per megabase in the analysed genomic sequence. This implies that NSCLC patients with *EGFR*-sensitive mutations and high PD-L1 expression may harbor a higher burden of genetic mutations, potentially influencing tumor-related pathways. This correlation may also contribute to the relatively unfavorable prognosis observed in patients with *EGFR*-sensitive mutations and high TMB [12], as well as in those with *EGFR*-sensitive mutations accompanied by high PD-L1 expression ^13^. Our mutation profile analysis demonstrated notable differences in the top 10 mutated genes and their mutation rates between the high and low PD-L1 expression or TMB-H and TMB-L groups. Comparative analysis of mutations further supports these distinctions, suggesting potential variations in tumor signaling pathways between these groups. A more in-depth pathway mutation analysis revealed that *EGFR*-sensitive mutations, whether accompanied by high TMB or high PD-L1 expression, exhibit a higher mutation rate in the PI3K signaling pathway.

The PI3K pathway plays an important role in tumor development and progression. It is a signaling pathway involved in the regulation of multiple biological processes such as cell growth, survival, proliferation, and metabolism. Aberrant activation of the PI3K pathway is closely associated with the occurrence and progression of various types of cancer. Studies have shown that aberrant activation of the PI3K pathway can lead to increased tumor cell growth, inhibition of apoptosis, promotion of angiogenesis, and enhanced metastasis and invasion capabilities [21–23]. This is because the activation of the PI3K pathway can promote cell cycle progression, enhance signaling for cell proliferation and growth, inhibit programmed cell death, and facilitate tumor cell invasion and metastasis by regulating the cell cytoskeleton and matrix metalloproteinase expression. Our results confirm that the PI3K pathway is often activated through *PIK3CA* mutations/amplifications and *PTEN* loss, which aligns with the established knowledge. Contrary to the mutual exclusivity observed in most oncogenic driver gene mutations, *PIK3CA* mutations often cooccur with other oncogenic driver gene mutations in NSCLC. In the AURA3 study [24], the incidence of *PIK3CA* amplification/mutation in patients with acquired resistance to second-line treatment with osimertinib was 5%, with two patients having concurrent *PIK3CA* amplification and *HER2* amplification. Among patients who developed resistance to first-line treatment with osimertinib [9], 7% were found to have *PIK3CA* mutations, with the most common being the E545K mutation (4%), followed by E453K and H1047R. These studies suggest that activation of the PI3K pathway may be the reason for the poor response to *EGFR*-TKIs in patients with *EGFR*-sensitive mutations accompanied by high TMB or high PD-L1 expression.

It should be noted that our study has some limitations. First, the number of patients with *EGFR*-sensitive mutations accompanied by high PD-L1 expression or high TMB in our study was relatively small, which may introduce bias, and further expansion of the sample size is needed. Second, we only analysed pathway-related gene mutations, and further validation is needed to determine whether the mutations truly affect pathway expression. Most importantly, we lacked relevant follow-up data and *EGFR*-TKI medication data, and further validation of our conclusions is needed by considering the patient’s prognosis.

In summary, our research found that NSCLC patients with *EGFR*-sensitive mutations accompanied by high expression of PD-L1 or high TMB values may have a higher frequency of abnormal activation in the PI3K pathway. This, in turn, may lead to a poorer response to *EGFR*-TKI treatment compared with other types of patients. Our findings provide an understanding of the resistance mechanisms in these patients and offer new insights and directions for precise treatment.

### Electronic supplementary material

Below is the link to the electronic supplementary material.

**Supplementary Material 1: Figure S1:** The relationship between TMB and MSI. **Figure S2:** Mutation overview of collected samples. **Figure S3:** Pathway mutation differential analysis in high or nonhigh PD-L1 expression group. **Figure S4:** Pathway mutation differential analysis in high or low TMB value group. **Figure S5:** Distribution of mutations in the PIK3CA and PTEN genes. **Table S1:** Detailed information for each patient. **Table S2:** The gene list of AllNGS-Panel 639. **Table S3:** Association between TMB status and clinical features. **Table S4:** Differential analysis of mutations in signaling pathways related to EGFR-sensitive mutations or high PD-L1 expression. **Table S5:** Differential analysis of mutations in signaling pathways related to EGFR-sensitive mutations or TMB-H

## Data Availability

The data supporting this study’s findings are available on request from the corresponding author. The data are not publicly available due to privacy or ethical restrictions.

## Abbreviations

E19del: exon 19 deletion
InDel: insertion or deletion
LUAD: lung adenocarcinoma
LUSC: lung squamous cell carcinoma
MSI: Microsatellite instability
NGS: next-generation sequencing
NSCLC: non-small cell lung cancer
PD-L1: programmed death-ligand 1
PI3K: phosphoInositide 3-kinase
SNP: single nucleotide polymorphism
SNV: single nucleotide variation
TKI: tyrosine kinase inhibitor
TMB: tumor mutational burden
TPS: tumor proportion score
