# Supplementary material for: Unraveling EGFR-TKI resistance in lung cancer with high PD-L1 or TMB in EGFR-sensitive mutations
Source: Respir Res. 2024 Jan 18;25:40. doi: 10.1186/s12931-023-02656-3 (PMC10797755; doi:10.1186/s12931-023-02656-3)
Supplement: Supplementary file 1 — Supplementary Material 1: Figure S1: The relationship between TMB and MSI. Figure S2: Mutation overview of collected samples. Figure S3: Pathway mutation differential analysis in high or nonhigh PD-L1 expression group. Figure S4: Pathway mutation differential analysis in high or low TMB value group. Figure S5: Distribution of mutations in the PIK3CA and PTEN genes. Table S1: Detailed information for each patient. Table S2: The gene list of AllNGS-Panel 639. Table S3: Association between TMB status and clinical features. Table S4: Differential analysis of mutations in signaling pathways related to EGFR-sensitive mutations or high PD-L1 expression. Table S5: Differential analysis of mutations in signaling pathways related to EGFR-sensitive mutations or TMB-H [file 12931_2023_2656_MOESM1_ESM.docx]

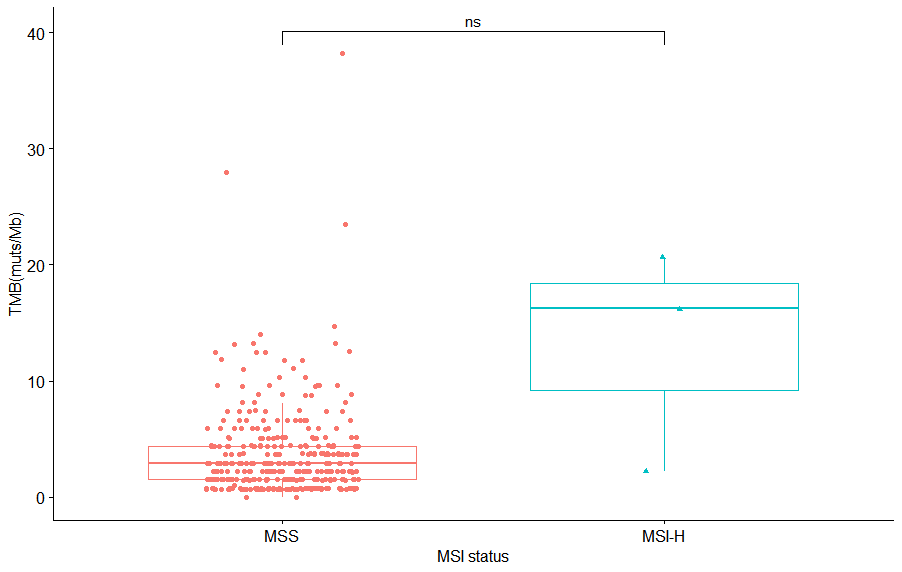


**Figure S1 The relationship between TMB and MSI**

“ns” indicates P > 0.05, Wilcox test


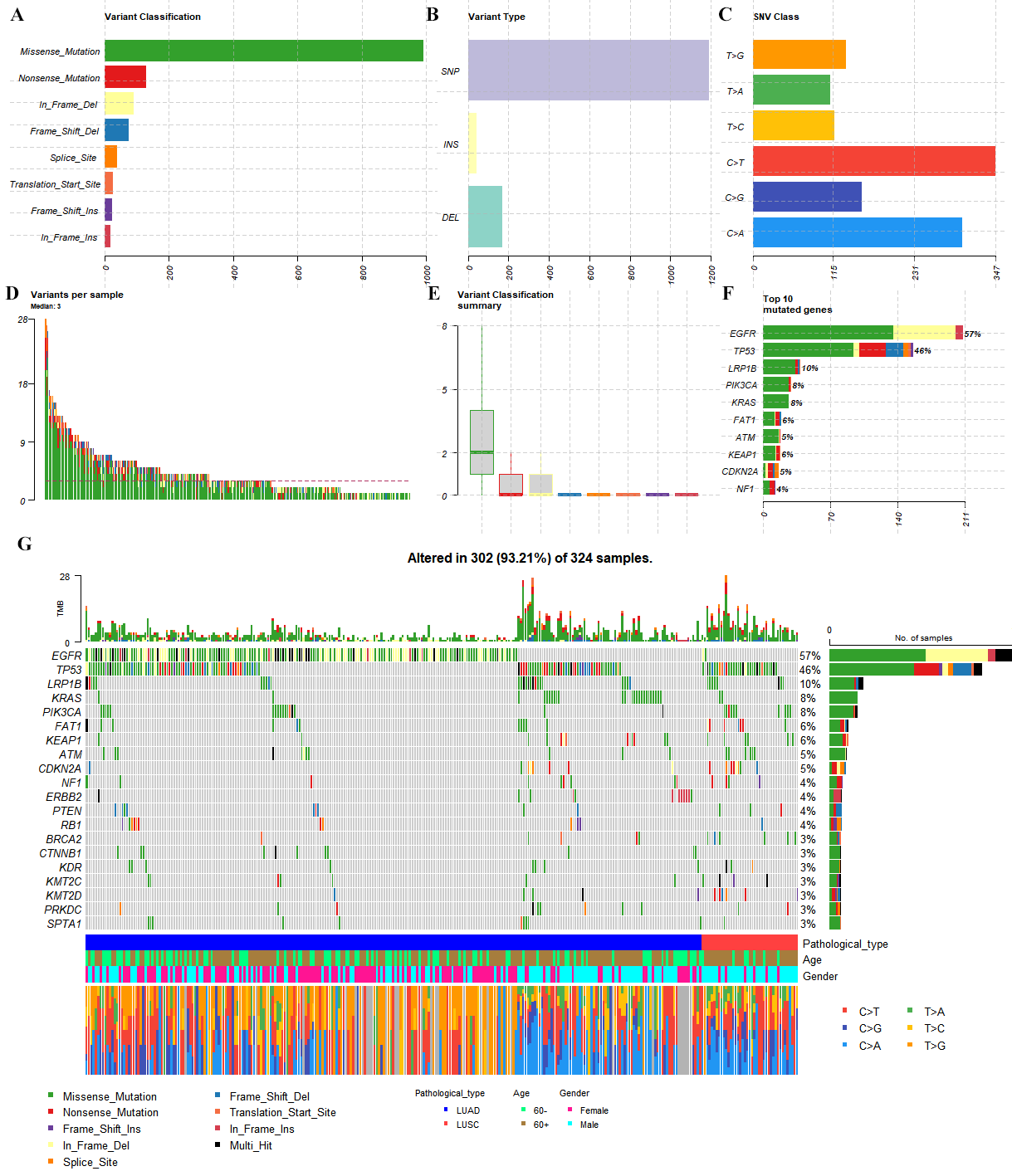


**Figure S2 Mutation overview of collected samples**

(A) The variant classification, (B)variant type, and (C) SNV class of mutated genes involved in NSCLC tumors; (D) variants in each sample; (E) summary of variant classification ; (F) mutation types of the top 10 genes; (G) the water-fall diagram indicates the top 20 mutated genes and their variant types in SCLC tissues based on NGS sequencing data from collected samples.


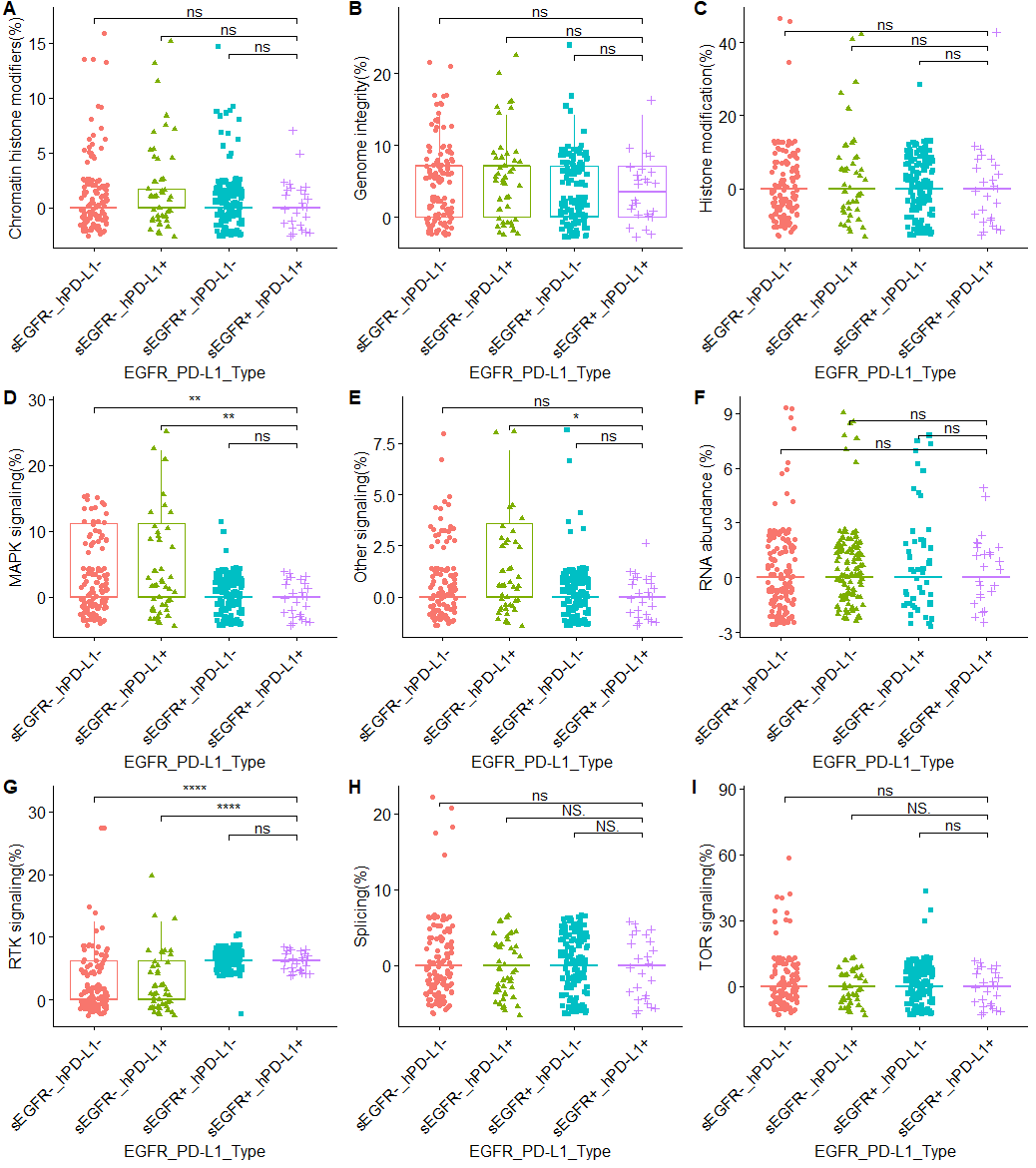


**Fig S3 Pathway mutation differential analysis in high or nonhigh PD-L1 expression group**

Differential analysis of signaling pathways: chromatin histone modifiers (A), genome integrity (B), histone modification (C), MAPK signaling (D), other signaling (E), RNA abundance (F), RTK signaling (G), splicing (H), and TOR signaling (I).

“ns”, “*”, “**” and “****” indicate P > 0.05, P < 0.05, P < 0.01, and P < 0.0001, Wilcox test

sEGFR-_hPD-L1-: group without EGFR E19del/L858R mutation and nonhigh PD-L1 expression (TPS < 50%);

sEGFR-_hPD-L1+: group without EGFR E19del/L858R mutation and high PD-L1 expression (TPS ≥ 50%);

sEGFR+_hPD-L1-: group with EGFR E19del/L858R mutation and nonhigh PD-L1 expression (TPS < 50%);

sEGFR+_hPD-L1+: group with EGFR E19del/L858R mutation and high PD-L1 expression (TPS ≥ 50%).


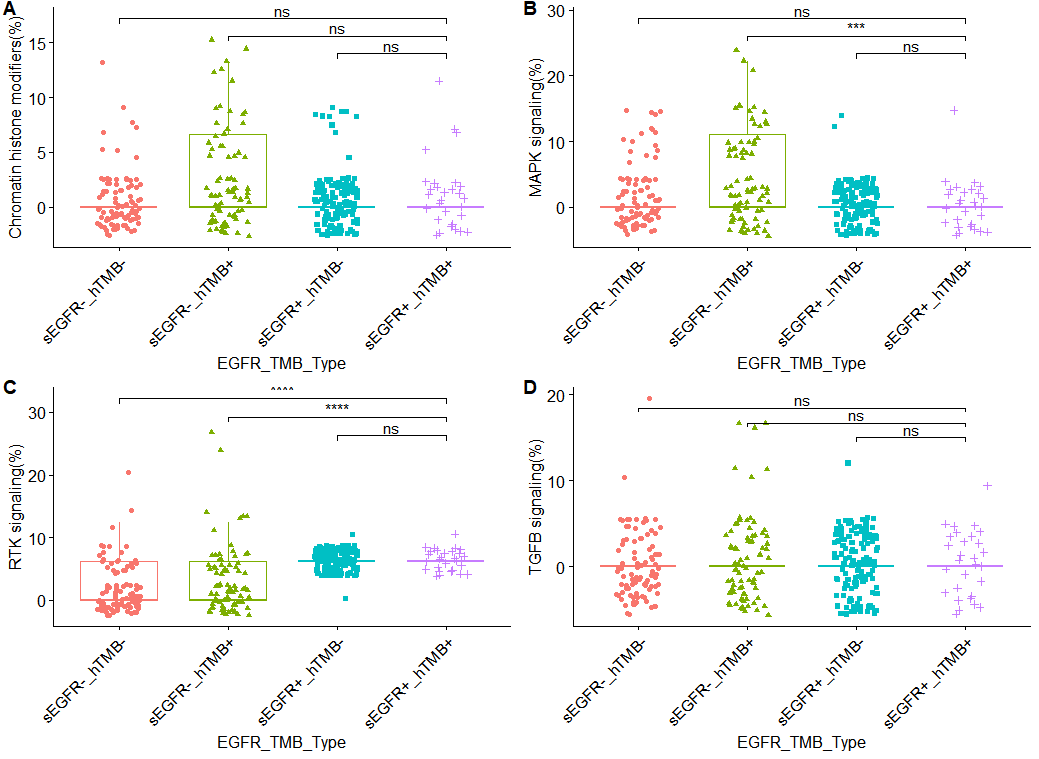


**Fig S4 Pathway mutation differential analysis in high or low TMB value group**

Differential analysis of signaling pathways: chromatin histone modifiers (A), MAPK signaling (B), RTK signaling (C), and TGFB signaling (D).

“ns”, “***” and “****” indicate P > 0.05, P < 0.001 and P < 0.0001, Wilcox test

sEGFR-_hTMB-: group without EGFR E19del/L858R mutation and low TMB value (TMB < 4.4 muts/Mb);

sEGFR-_hTMB+: group without EGFR E19del/L858R mutation and high TMB value (TMB ≥ 4.4 muts/Mb);

sEGFR+_hTMB-: group with EGFR E19del/L858R mutation and low TMB value (TMB < 4.4 muts/Mb);

sEGFR+_hPD-L1+: group with EGFR E19del/L858R mutation and high TMB value (TMB ≥ 4.4 muts/Mb).


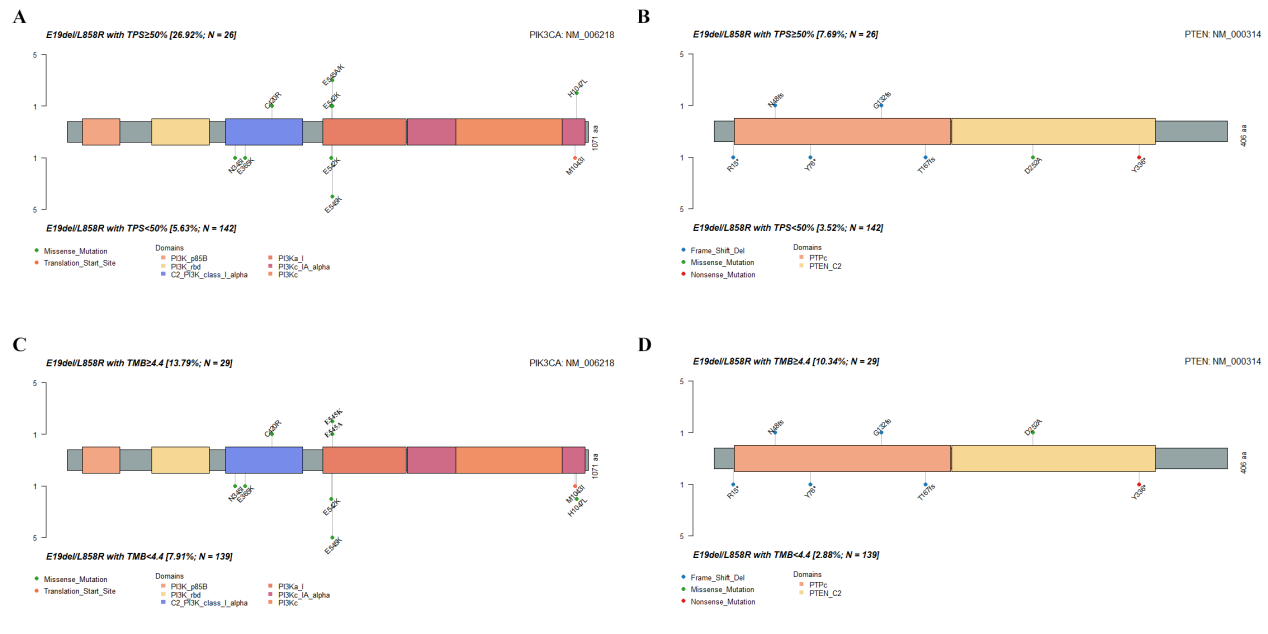


**Figure S5. Distribution of mutations in the *PIK3CA* and *PTEN* genes**

*PIK3CA* (A) and *PTEN* (B) mutations in lung cancer with *EGFR*-sensitive mutations and high (TPS ≥ 50%) and nonhigh (TPS < 50%) PD-L1 expression; *PIK3CA* (C) and *PTEN* (D) mutations in lung cancer with *EGFR*-sensitive mutations and high (TMB ≥ 4.4 muts/Mb) or low (TMB < 4.4 muts/Mb) TMB values.

**Table S1 Detailed information for each patient**

| Patient ID | **Pathological**  **type** | **Gender** | **Age** | **EGFR mutation** | **MSI score** | **TMB score**  **(muts/Mb)** | **PD-L1 expression score (TPS)** |
| --- | --- | --- | --- | --- | --- | --- | --- |
| P001 | Adenocarcinoma | Female | 64 | E19del | 4.54% | 1 | 1%-2% |
| P002 | Adenocarcinoma | Male | 57 | E19del | 4.54% | 3.7 | ＜1% |
| P003 | Adenocarcinoma | Female | 64 | L858R | 0.00% | 0.7 | ＜1% |
| P004 | Adenocarcinoma | Female | 32 | WT | 4.54% | 2.2 | ＜1% |
| P005 | Adenocarcinoma | Male | 72 | E19del | 0.00% | 3.7 | ＜1% |
| P006 | Squamous cell carcinoma | Male | 69 | WT | 4.54% | 2.9 | ＜1% |
| P007 | Adenocarcinoma | Female | 32 | E19del | 0.00% | 0.7 | ＜1% |
| P008 | Adenocarcinoma | Female | 56 | L858R | 0.00% | 0.7 | ＜1% |
| P009 | Adenocarcinoma | Female | 76 | L858R | 13.64% | 1.5 | 10% |
| P010 | Adenocarcinoma | Female | 45 | WT | 0.00% | 0.7 | ＜1% |
| P011 | Adenocarcinoma | Male | 50 | WT | 4.54% | 2.2 | 80% |
| P012 | Adenocarcinoma | Male | 63 | WT | 13.64% | 3.7 | ＜1% |
| P013 | Adenocarcinoma | Female | 70 | WT | 4.54% | 2.2 | ＜1% |
| P014 | Adenocarcinoma | Male | 59 | WT | 0.00% | 1.5 | 40% |
| P015 | Squamous cell carcinoma | Male | 72 | WT | 5.56% | 0.7 | ＜1% |
| P016 | Adenocarcinoma | Male | 62 | WT | 13.33% | 27.9 | ＜1% |
| P017 | Adenocarcinoma | Female | 62 | E19del | 4.54% | 0.7 | ＜1% |
| P018 | Adenocarcinoma | Male | 73 | WT | 9.09% | 4.4 | ＜1% |
| P019 | Adenocarcinoma | Male | 68 | WT | 9.09% | 1.5 | ＜1% |
| P020 | Squamous cell carcinoma | Male | 53 | WT | 9.09% | 13.2 | 90% |
| P021 | Adenocarcinoma | Male | 68 | L858R | 4.54% | 0.7 | ＜1% |
| P022 | Adenocarcinoma | Male | 86 | L858R | 0.00% | 1.5 | ＜1% |
| P023 | Adenocarcinoma | Female | 59 | L858R | 9.09% | 2.9 | ＜1% |
| P024 | Adenocarcinoma | Female | 74 | E19del | 4.54% | 1.5 | ＜1% |
| P025 | Adenocarcinoma | Female | 52 | E19del | 5.56% | 0 | ＜1% |
| P026 | Adenocarcinoma | Female | 57 | E19del | 5.56% | 1.5 | ＜1% |
| P027 | Adenocarcinoma | Female | 43 | WT | 0.00% | 0.7 | ＜1% |
| P028 | Squamous cell carcinoma | Male | 52 | WT | 0.00% | 3.7 | ＜1% |
| P029 | Squamous cell carcinoma | Male | 30 | E19del | 5.88% | 2.9 | 5% |
| P030 | Adenocarcinoma | Female | 68 | L858R | 18.18% | 1.5 | ＜1% |
| P031 | Adenocarcinoma | Female | 76 | WT | 9.09% | 0.7 | 5% |
| P032 | Squamous cell carcinoma | Male | 78 | WT | 18.18% | 3.7 | ＜1% |
| P033 | Squamous cell carcinoma | Male | 64 | WT | 4.54% | 14.7 | 70% |
| P034 | Adenocarcinoma | Female | 68 | E19del | 9.09% | 0.7 | ＜1% |
| P035 | Adenocarcinoma | Female | 68 | E19del | 4.54% | 1.5 | ＜1% |
| P036 | Adenocarcinoma | Female | 57 | E19del | 4.54% | 2.2 | ＜1% |
| P037 | Adenocarcinoma | Male | 70 | E19del | 4.54% | 7.4 | 40% |
| P038 | Adenocarcinoma | Female | 64 | L858R | 4.54% | 6.6 | ＜1% |
| P039 | Adenocarcinoma | Female | 30 | WT | 0.00% | 0.7 | 98% |
| P040 | Adenocarcinoma | Female | 82 | other | 0.00% | 8.8 | ＜1% |
| P041 | Adenocarcinoma | Female | 33 | WT | 13.64% | 0.7 | ＜1% |
| P042 | Adenocarcinoma | Female | 51 | WT | 22.22% | 2.9 | ＜1% |
| P043 | Adenocarcinoma | Male | 74 | WT | 0.00% | 0.7 | ＜1% |
| P044 | Adenocarcinoma | Male | 51 | L858R | 0.00% | 1.5 | ＜1% |
| P045 | Adenocarcinoma | Male | 46 | WT | 0.00% | 5.9 | 98% |
| P046 | Adenocarcinoma | Male | 67 | E19del | 9.09% | 0.7 | ＜1% |
| P047 | Adenocarcinoma | Female | 58 | WT | 11.11% | 1.5 | 10% |
| P048 | Adenocarcinoma | Female | 48 | E19del | 4.54% | 0.7 | ＜1% |
| P049 | Adenocarcinoma | Male | 82 | WT | 9.09% | 3.7 | ＜1% |
| P050 | Squamous cell carcinoma | Male | 66 | WT | 0.00% | 4.4 | 8% |
| P051 | Adenocarcinoma | Female | 25 | other | 9.09% | 0.7 | 2% |
| P052 | Adenocarcinoma | Female | 65 | L858R | 9.09% | 1.5 | 2% |
| P053 | Adenocarcinoma | Male | 71 | WT | 4.54% | 7.4 | ＜1% |
| P054 | Adenocarcinoma | Male | 65 | E19del | 4.54% | 7.4 | 90% |
| P055 | Adenocarcinoma | Female | 32 | WT | 9.09% | 0.7 | ＜1% |
| P056 | Adenocarcinoma | Female | 77 | E19del | 9.09% | 0.7 | 5% |
| P057 | Adenocarcinoma | Female | 59 | E19del | 22.73% | 3.7 | ＜1% |
| P058 | Adenocarcinoma | Female | 69 | E19del | 0.00% | 0.7 | ＜1% |
| P059 | Adenocarcinoma | Female | 57 | E19del | 13.64% | 9.5 | ＜1% |
| P060 | Adenocarcinoma | Female | 60 | L858R | 0.00% | 2.2 | ＜1% |
| P061 | Adenocarcinoma | Male | 49 | L858R | 9.09% | 0.7 | ＜1% |
| P062 | Adenocarcinoma | Male | 56 | E19del | 9.09% | 1.5 | ＜1% |
| P063 | Adenocarcinoma | Female | 41 | WT | 4.54% | 2.2 | 40% |
| P064 | Adenocarcinoma | Male | 73 | WT | 27.27% | 1.5 | ＜1% |
| P065 | Adenocarcinoma | Female | 68 | L858R | 13.64% | 0.7 | ＜1% |
| P066 | Adenocarcinoma | Male | 66 | WT | 9.09% | 5.9 | ＜1% |
| P067 | Adenocarcinoma | Male | 60 | L858R | 9.09% | 4.4 | 5% |
| P068 | Adenocarcinoma | Male | 76 | WT | 13.64% | 5.1 | ＜1% |
| P069 | Adenocarcinoma | Male | 74 | L858R | 9.09% | 1.5 | 25% |
| P070 | Adenocarcinoma | Female | 55 | WT | 4.54% | 0.7 | 10% |
| P071 | Adenocarcinoma | Male | 58 | WT | 28.57% | 5.9 | ＜1% |
| P072 | Squamous cell carcinoma | Male | 74 | WT | 4.54% | 5.1 | ＜1% |
| P073 | Adenocarcinoma | Female | 58 | other | 18.18% | 3.7 | 95% |
| P074 | Adenocarcinoma | Female | 36 | WT | 9.09% | 0.7 | 90% |
| P075 | Adenocarcinoma | Female | 69 | L858R | 0.00% | 0.7 | 5% |
| P076 | Adenocarcinoma | Female | 58 | L858R | 18.18% | 1.5 | 1%-2% |
| P077 | Adenocarcinoma | Male | 60 | WT | 4.54% | 9.6 | 75% |
| P078 | Squamous cell carcinoma | Female | 55 | WT | 0.00% | 11.8 | 1%-2% |
| P079 | Adenocarcinoma | Female | 80 | other | 4.54% | 2.2 | 2% |
| P080 | Adenocarcinoma | Male | 64 | WT | 13.64% | 1.5 | ＜1% |
| P081 | Adenocarcinoma | Male | 69 | WT | 13.64% | 9.6 | 95% |
| P082 | Adenocarcinoma | Male | 72 | WT | 4.54% | 11 | 95% |
| P083 | Adenocarcinoma | Female | 56 | WT | 13.64% | 2.2 | ＜1% |
| P084 | Squamous cell carcinoma | Female | 75 | WT | 36.36% | 16.2 | 5% |
| P085 | Adenocarcinoma | Female | 57 | WT | 22.73% | 9.6 | 95% |
| P086 | Adenocarcinoma | Male | 65 | WT | 22.73% | 12.5 | ＜1% |
| P087 | Adenocarcinoma | Male | 66 | WT | 22.73% | 3.7 | ＜1% |
| P088 | Adenocarcinoma | Male | 44 | WT | 40.91% | 20.6 | 35% |
| P089 | Adenocarcinoma | Male | 68 | L858R | 13.64% | 3.7 | 5% |
| P090 | Adenocarcinoma | Male | 43 | E19del | 0.00% | 0.7 | ＜1% |
| P091 | Adenocarcinoma | Female | 47 | E19del | 4.54% | 5.9 | 98% |
| P092 | Adenocarcinoma | Male | 46 | L858R | 9.09% | 0.7 | ＜1% |
| P093 | Adenocarcinoma | Female | 56 | other | 9.09% | 0.7 | 55% |
| P094 | Adenocarcinoma | Female | 59 | L858R | 4.54% | 4.4 | 15% |
| P095 | Adenocarcinoma | Female | 52 | other | 9.09% | 5.1 | ＜1% |
| P096 | Squamous cell carcinoma | Male | 68 | WT | 27.27% | 13.2 | 50% |
| P097 | Adenocarcinoma | Female | 56 | E19del | 4.54% | 2.2 | ＜1% |
| P098 | Adenocarcinoma | Male | 51 | L858R | 4.54% | 1.5 | 3% |
| P099 | Squamous cell carcinoma | Female | 55 | WT | 9.09% | 5.1 | 98% |
| P100 | Adenocarcinoma | Female | 69 | L858R | 0.00% | 0.7 | ＜1% |
| P101 | Adenocarcinoma | Male | 69 | WT | 0.00% | 10.3 | 10% |
| P102 | Squamous cell carcinoma | Male | 70 | WT | 0.00% | 5.9 | 98% |
| P103 | Adenocarcinoma | Male | 69 | WT | 22.73% | 2.9 | 80% |
| P104 | Squamous cell carcinoma | Male | 64 | WT | 0.00% | 2.9 | 40% |
| P105 | Squamous cell carcinoma | Male | 68 | WT | 4.54% | 4.4 | 80% |
| P106 | Adenocarcinoma | Female | 70 | WT | 4.54% | 38.2 | ＜1% |
| P107 | Adenocarcinoma | Female | 77 | WT | 9.09% | 6.6 | 98% |
| P108 | Adenocarcinoma | Female | 60 | E19del | 0.00% | 2.9 | 65% |
| P109 | Adenocarcinoma | Female | 58 | L858R | 4.54% | 0.7 | ＜1% |
| P110 | Adenocarcinoma | Male | 74 | L858R | 18.18% | 4.4 | ＜1% |
| P111 | Adenocarcinoma | Male | 64 | WT | 0.00% | 12.5 | 50% |
| P112 | Adenocarcinoma | Female | 66 | WT | 0.00% | 2.2 | ＜1% |
| P113 | Squamous cell carcinoma | Male | 72 | WT | 0.00% | 11 | 98% |
| P114 | Adenocarcinoma | Female | 54 | other | 36.36% | 2.2 | 90% |
| P115 | Adenocarcinoma | Female | 55 | WT | 0.00% | 0.7 | 35% |
| P116 | Adenocarcinoma | Male | 48 | L858R | 4.54% | 2.2 | 3% |
| P117 | Squamous cell carcinoma | Female | 71 | WT | 4.54% | 23.5 | 3% |
| P118 | Adenocarcinoma | Male | 61 | E19del | 9.09% | 2.9 | ＜1% |
| P119 | Adenocarcinoma | Male | 64 | WT | 18.18% | 7.4 | ＜1% |
| P120 | Adenocarcinoma | Male | 66 | L858R | 4.54% | 4.4 | 55% |
| P121 | Adenocarcinoma | Female | 74 | E19del | 4.54% | 2.2 | 10% |
| P122 | Adenocarcinoma | Male | 74 | WT | 13.64% | 6.6 | 98% |
| P123 | Adenocarcinoma | Male | 57 | WT | 0.00% | 0.7 | ＜1% |
| P124 | Adenocarcinoma | Male | 51 | E19del | 4.54% | 0.7 | 70% |
| P125 | Squamous cell carcinoma | Male | 71 | WT | 4.54% | 5.9 | 45% |
| P126 | Adenocarcinoma | Male | 57 | L858R | 13.64% | 3.7 | 97% |
| P127 | Adenocarcinoma | Female | 62 | E19del | 0.00% | 0.7 | 2% |
| P128 | Adenocarcinoma | Male | 49 | WT | 5.26% | 4.4 | 85% |
| P129 | Adenocarcinoma | Female | 54 | E19del | 0.00% | 0.7 | 3% |
| P130 | Adenocarcinoma | Female | 55 | L858R | 0.00% | 1.5 | ＜1% |
| P131 | Adenocarcinoma | Male | 79 | WT | 18.18% | 3.7 | ＜1% |
| P132 | Adenocarcinoma | Male | 46 | L858R | 0.00% | 2.9 | ＜1% |
| P133 | Adenocarcinoma | Female | 79 | WT | 4.54% | 2.2 | ＜1% |
| P134 | Adenocarcinoma | Female | 76 | E19del | 0.00% | 2.2 | ＜1% |
| P135 | Adenocarcinoma | Male | 51 | E19del | 0.00% | 0.7 | ＜1% |
| P136 | Adenocarcinoma | Female | 65 | WT | 4.54% | 0.7 | 35% |
| P137 | Adenocarcinoma | Female | 44 | WT | 0.00% | 2.2 | 99% |
| P138 | Adenocarcinoma | Male | 55 | E19del | 0.00% | 1.5 | 5% |
| P139 | Adenocarcinoma | Male | 76 | WT | 4.54% | 7.4 | 85% |
| P140 | Adenocarcinoma | Female | 82 | E19del | 0.00% | 2.2 | ＜1% |
| P141 | Adenocarcinoma | Male | 78 | WT | 4.54% | 3.7 | ＜1% |
| P142 | Adenocarcinoma | Female | 56 | L858R | 0.00% | 1.5 | 5% |
| P143 | Squamous cell carcinoma | Female | 46 | WT | 4.54% | 4.4 | 10% |
| P144 | Squamous cell carcinoma | Male | 75 | WT | 0.00% | 12.5 | ＜1% |
| P145 | Adenocarcinoma | Male | 67 | other | 0.00% | 5.9 | ＜1% |
| P146 | Adenocarcinoma | Male | 59 | L858R | 4.54% | 3.7 | 2% |
| P147 | Adenocarcinoma | Female | 58 | other | 0.00% | 0.7 | 75% |
| P148 | Adenocarcinoma | Female | 42 | WT | 0.00% | 1.5 | ＜1% |
| P149 | Adenocarcinoma | Male | 71 | L858R | 0.00% | 0.7 | ＜1% |
| P150 | Adenocarcinoma | Male | 76 | WT | 0.00% | 12.5 | ＜1% |
| P151 | Adenocarcinoma | Male | 61 | L858R | 0.00% | 4.4 | ＜1% |
| P152 | Adenocarcinoma | Male | 68 | E19del | 0.00% | 2.2 | ＜1% |
| P153 | Adenocarcinoma | Male | 78 | WT | 0.00% | 6.6 | ＜1% |
| P154 | Squamous cell carcinoma | Male | 74 | WT | 0.00% | 5.1 | ＜1% |
| P155 | Adenocarcinoma | Female | 51 | L858R | 9.09% | 0.7 | ＜1% |
| P156 | Squamous cell carcinoma | Male | 73 | WT | 4.54% | 4.4 | 40% |
| P157 | Adenocarcinoma | Male | 76 | L858R | 0.00% | 1.5 | ＜1% |
| P158 | Adenocarcinoma | Male | 67 | L858R | 0.00% | 0.7 | ＜1% |
| P159 | Adenocarcinoma | Male | 57 | L858R | 4.54% | 3.7 | ＜1% |
| P160 | Adenocarcinoma | Male | 78 | L858R | 0.00% | 2.9 | 10% |
| P161 | Adenocarcinoma | Male | 46 | WT | 0.00% | 3.7 | ＜1% |
| P162 | Adenocarcinoma | Female | 79 | L858R | 0.00% | 2.9 | ＜1% |
| P163 | Adenocarcinoma | Male | 49 | other | 4.54% | 4.4 | ＜1% |
| P164 | Adenocarcinoma | Female | 58 | WT | 4.54% | 0.7 | ＜1% |
| P165 | Adenocarcinoma | Male | 49 | L858R | 0.00% | 1.5 | ＜1% |
| P166 | Adenocarcinoma | Male | 63 | L858R | 4.54% | 2.2 | ＜1% |
| P167 | Squamous cell carcinoma | Male | 63 | WT | 18.18% | 2.9 | ＜1% |
| P168 | Adenocarcinoma | Male | 32 | E19del | 13.64% | 0.7 | 5% |
| P169 | Squamous cell carcinoma | Male | 72 | WT | 9.09% | 8.8 | ＜1% |
| P170 | Adenocarcinoma | Male | 64 | other | 0.00% | 6.6 | ＜1% |
| P171 | Squamous cell carcinoma | Male | 64 | other | 0.00% | 4.4 | 5% |
| P172 | Adenocarcinoma | Female | 48 | WT | 0.00% | 1.5 | 3% |
| P173 | Adenocarcinoma | Female | 64 | L858R | 9.09% | 1.5 | ＜1% |
| P174 | Squamous cell carcinoma | Male | 66 | WT | 4.54% | 10.3 | ＜1% |
| P175 | Adenocarcinoma | Female | 59 | L858R | 4.54% | 1.5 | 10% |
| P176 | Adenocarcinoma | Male | 72 | E19del | 0.00% | 1.5 | 70% |
| P177 | Adenocarcinoma | Female | 59 | E19del | 4.54% | 3.7 | 98% |
| P178 | Adenocarcinoma | Female | 64 | other | 0.00% | 0.7 | ＜1% |
| P179 | Adenocarcinoma | Female | 68 | E19del | 0.00% | 8.1 | 45% |
| P180 | Adenocarcinoma | Female | 65 | L858R | 4.54% | 3.7 | ＜1% |
| P181 | Adenocarcinoma | Female | 57 | E19del | 4.54% | 0.7 | ＜1% |
| P182 | Adenocarcinoma | Female | 67 | L858R | 4.54% | 3.7 | ＜1% |
| P183 | Adenocarcinoma | Male | 54 | L858R | 0.00% | 4.4 | 3% |
| P184 | Adenocarcinoma | Female | 60 | L858R | 0.00% | 2.9 | ＜1% |
| P185 | Adenocarcinoma | Female | 45 | E19del | 0.00% | 2.9 | 1%-2% |
| P186 | Adenocarcinoma | Female | 48 | L858R | 4.54% | 2.9 | 5% |
| P187 | Adenocarcinoma | Female | 60 | L858R | 0.00% | 3.7 | ＜1% |
| P188 | Adenocarcinoma | Male | 56 | L858R | 4.54% | 1.5 | 8% |
| P189 | Adenocarcinoma | Male | 60 | WT | 4.54% | 8.8 | 98% |
| P190 | Adenocarcinoma | Female | 46 | other | 0.00% | 1.5 | 80% |
| P191 | Adenocarcinoma | Female | 60 | L858R | 4.54% | 4.4 | 55% |
| P192 | Adenocarcinoma | Female | 68 | L858R | 0.00% | 1.5 | 80% |
| P193 | Adenocarcinoma | Male | 68 | L858R | 0.00% | 0.7 | ＜1% |
| P194 | Adenocarcinoma | Male | 57 | E19del | 4.54% | 2.2 | ＜1% |
| P195 | Adenocarcinoma | Male | 73 | L858R | 13.64% | 4.4 | 98% |
| P196 | Adenocarcinoma | Female | 64 | E19del | 0.00% | 0.7 | ＜1% |
| P197 | Adenocarcinoma | Male | 51 | L858R | 0.00% | 2.2 | 60% |
| P198 | Adenocarcinoma | Female | 58 | WT | 0.00% | 1.5 | 55% |
| P199 | Adenocarcinoma | Female | 74 | other | 0.00% | 2.2 | ＜1% |
| P200 | Adenocarcinoma | Male | 46 | WT | 0.00% | 2.2 | ＜1% |
| P201 | Squamous cell carcinoma | Male | 72 | other | 4.54% | 2.9 | ＜1% |
| P202 | Adenocarcinoma | Female | 62 | WT | 0.00% | 2.9 | 95% |
| P203 | Adenocarcinoma | Female | 73 | WT | 0.00% | 14 | 98% |
| P204 | Adenocarcinoma | Male | 83 | WT | 0.00% | 8.1 | ＜1% |
| P205 | Adenocarcinoma | Male | 60 | other | 0.00% | 4.4 | ＜1% |
| P206 | Squamous cell carcinoma | Female | 70 | WT | 0.00% | 6.6 | ＜1% |
| P207 | Adenocarcinoma | Male | 54 | WT | 4.54% | 2.2 | 30% |
| P208 | Adenocarcinoma | Male | 64 | WT | 4.54% | 5.1 | 2% |
| P209 | Adenocarcinoma | Female | 56 | E19del | 4.54% | 0.7 | 95% |
| P210 | Adenocarcinoma | Male | 31 | WT | 0.00% | 0.7 | ＜1% |
| P211 | Adenocarcinoma | Female | 49 | E19del | 0.00% | 0.7 | ＜1% |
| P212 | Adenocarcinoma | Female | 76 | L858R | 0.00% | 3.7 | 98% |
| P213 | Adenocarcinoma | Male | 68 | L858R | 0.00% | 2.2 | ＜1% |
| P214 | Adenocarcinoma | Female | 62 | L858R | 0.00% | 1.5 | ＜1% |
| P215 | Adenocarcinoma | Female | 66 | L858R | 0.00% | 3.7 | 30% |
| P216 | Adenocarcinoma | Female | 75 | other | 0.00% | 1.5 | ＜1% |
| P217 | Adenocarcinoma | Female | 62 | L858R | 4.54% | 0 | ＜1% |
| P218 | Adenocarcinoma | Female | 68 | WT | 0.00% | 2.9 | 8% |
| P219 | Adenocarcinoma | Male | 73 | WT | 18.18% | 0.7 | ＜1% |
| P220 | Adenocarcinoma | Male | 80 | L858R | 4.54% | 9.6 | ＜1% |
| P221 | Adenocarcinoma | Female | 57 | E19del | 4.54% | 2.9 | 1%-2% |
| P222 | Adenocarcinoma | Female | 60 | L858R | 0.00% | 0.7 | ＜1% |
| P223 | Adenocarcinoma | Female | 74 | WT | 4.54% | 0.7 | ＜1% |
| P224 | Adenocarcinoma | Male | 61 | L858R | 0.00% | 2.2 | ＜1% |
| P225 | Adenocarcinoma | Male | 75 | WT | 0.00% | 2.2 | ＜1% |
| P226 | Adenocarcinoma | Female | 71 | WT | 0.00% | 5.1 | 40% |
| P227 | Squamous cell carcinoma | Male | 61 | other | 0.00% | 8.8 | 90% |
| P228 | Adenocarcinoma | Female | 55 | WT | 0.00% | 0.7 | 99% |
| P229 | Adenocarcinoma | Male | 67 | WT | 0.00% | 5.1 | 1%-2% |
| P230 | Adenocarcinoma | Male | 60 | WT | 0.00% | 2.2 | 35% |
| P231 | Adenocarcinoma | Female | 67 | WT | 0.00% | 0.7 | 98% |
| P232 | Squamous cell carcinoma | Female | 58 | WT | 0.00% | 6.6 | 90% |
| P233 | Adenocarcinoma | Female | 63 | L858R | 4.54% | 5.9 | ＜1% |
| P234 | Adenocarcinoma | Female | 59 | WT | 4.54% | 0.7 | 2% |
| P235 | Adenocarcinoma | Male | 62 | L858R | 0.00% | 2.9 | 80% |
| P236 | Adenocarcinoma | Female | 65 | L858R | 9.09% | 2.9 | ＜1% |
| P237 | Adenocarcinoma | Male | 43 | WT | 0.00% | 0.7 | 65% |
| P238 | Adenocarcinoma | Female | 59 | E19del | 0.00% | 1.5 | ＜1% |
| P239 | Adenocarcinoma | Female | 70 | L858R | 0.00% | 2.9 | ＜1% |
| P240 | Adenocarcinoma | Female | 48 | L858R | 0.00% | 3.7 | 15% |
| P241 | Adenocarcinoma | Female | 65 | L858R | 0.00% | 3.7 | 8% |
| P242 | Adenocarcinoma | Female | 52 | E19del | 0.00% | 4.4 | 2% |
| P243 | Adenocarcinoma | Male | 53 | WT | 4.54% | 4.4 | ＜1% |
| P244 | Adenocarcinoma | Male | 70 | other | 4.54% | 2.9 | 30% |
| P245 | Adenocarcinoma | Male | 73 | L858R | 4.54% | 2.9 | ＜1% |
| P246 | Adenocarcinoma | Male | 59 | other | 4.54% | 2.2 | 95% |
| P247 | Adenocarcinoma | Female | 67 | E19del | 4.54% | 1.5 | 95% |
| P248 | Adenocarcinoma | Female | 71 | E19del | 9.09% | 5.1 | 45% |
| P249 | Adenocarcinoma | Male | 36 | E19del | 0.00% | 2.2 | 90% |
| P250 | Squamous cell carcinoma | Male | 53 | WT | 0.00% | 7.4 | ＜1% |
| P251 | Adenocarcinoma | Male | 70 | other | 0.00% | 5.1 | 98% |
| P252 | Adenocarcinoma | Female | 53 | other | 0.00% | 2.9 | ＜1% |
| P253 | Adenocarcinoma | Female | 47 | E19del | 0.00% | 0.7 | ＜1% |
| P254 | Adenocarcinoma | Male | 51 | WT | 4.54% | 8.8 | ＜1% |
| P255 | Adenocarcinoma | Male | 67 | WT | 0.00% | 6.6 | 99% |
| P256 | Adenocarcinoma | Female | 67 | WT | 0.00% | 2.2 | 80% |
| P257 | Squamous cell carcinoma | Male | 65 | WT | 0.00% | 2.9 | ＜1% |
| P258 | Adenocarcinoma | Female | 43 | WT | 0.00% | 2.2 | 5% |
| P259 | Squamous cell carcinoma | Male | 70 | WT | 0.00% | 3.7 | ＜1% |
| P260 | Adenocarcinoma | Female | 75 | L858R | 4.54% | 2.9 | ＜1% |
| P261 | Adenocarcinoma | Male | 73 | L858R | 9.09% | 2.9 | 2% |
| P262 | Adenocarcinoma | Male | 72 | L858R | 4.54% | 0.7 | ＜1% |
| P263 | Squamous cell carcinoma | Male | 77 | WT | 9.09% | 6.6 | ＜1% |
| P264 | Adenocarcinoma | Male | 76 | WT | 13.64% | 3.7 | 85% |
| P265 | Adenocarcinoma | Male | 66 | L858R | 4.54% | 0.7 | ＜1% |
| P266 | Adenocarcinoma | Male | 63 | WT | 9.09% | 0.7 | ＜1% |
| P267 | Adenocarcinoma | Male | 54 | E19del | 0.00% | 5.1 | 3% |
| P268 | Adenocarcinoma | Male | 76 | L858R | 0.00% | 5.1 | 95% |
| P269 | Adenocarcinoma | Female | 49 | L858R | 4.54% | 0.7 | ＜1% |
| P270 | Adenocarcinoma | Male | 69 | WT | 9.09% | 7.4 | ＜1% |
| P271 | Adenocarcinoma | Female | 66 | E19del | 0.00% | 2.9 | 25% |
| P272 | Adenocarcinoma | Male | 52 | L858R | 4.54% | 2.2 | ＜1% |
| P273 | Adenocarcinoma | Female | 68 | L858R | 9.09% | 6.6 | 60% |
| P274 | Adenocarcinoma | Male | 83 | L858R | 0.00% | 2.9 | ＜1% |
| P275 | Adenocarcinoma | Female | 40 | L858R | 0.00% | 0.7 | 2% |
| P276 | Squamous cell carcinoma | Male | 80 | WT | 0.00% | 5.9 | ＜1% |
| P277 | Adenocarcinoma | Female | 58 | L858R | 4.54% | 2.2 | 2% |
| P278 | Adenocarcinoma | Female | 68 | WT | 0.00% | 2.2 | 5% |
| P279 | Adenocarcinoma | Female | 50 | L858R | 0.00% | 3.7 | ＜1% |
| P280 | Adenocarcinoma | Female | 66 | L858R | 4.54% | 3.7 | ＜1% |
| P281 | Adenocarcinoma | Male | 47 | E19del | 9.09% | 11.8 | ＜1% |
| P282 | Adenocarcinoma | Female | 53 | E19del | 4.54% | 4.4 | ＜1% |
| P283 | Squamous cell carcinoma | Male | 64 | WT | 4.54% | 9.6 | 90% |
| P284 | Squamous cell carcinoma | Male | 64 | WT | 9.09% | 0.7 | 8% |
| P285 | Adenocarcinoma | Female | 47 | L858R | 0.00% | 0.7 | ＜1% |
| P286 | Adenocarcinoma | Female | 57 | E19del | 0.00% | 1.5 | 80% |
| P287 | Adenocarcinoma | Female | 78 | E19del | 4.54% | 1.5 | ＜1% |
| P288 | Adenocarcinoma | Female | 41 | WT | 4.54% | 0.7 | ＜1% |
| P289 | Adenocarcinoma | Female | 47 | WT | 0.00% | 0.7 | 95% |
| P290 | Adenocarcinoma | Female | 48 | E19del | 16.67% | 2.2 | 65% |
| P291 | Adenocarcinoma | Female | 63 | other | 4.17% | 2.9 | ＜1% |
| P292 | Squamous cell carcinoma | Male | 64 | WT | 0.00% | 4.4 | 10% |
| P293 | Adenocarcinoma | Female | 61 | WT | 0.00% | 2.9 | ＜1% |
| P294 | Adenocarcinoma | Female | 51 | WT | 0.00% | 1.5 | 45% |
| P295 | Adenocarcinoma | Male | 60 | WT | 4.17% | 2.2 | ＜1% |
| P296 | Adenocarcinoma | Male | 50 | WT | 4.17% | 5.1 | 3% |
| P297 | Adenocarcinoma | Female | 51 | other | 0.00% | 6.6 | ＜1% |
| P298 | Adenocarcinoma | Female | 70 | L858R | 0.00% | 1.5 | ＜1% |
| P299 | Adenocarcinoma | Female | 73 | L858R | 0.00% | 1.5 | ＜1% |
| P300 | Squamous cell carcinoma | Male | 68 | WT | 4.17% | 4.4 | ＜1% |
| P301 | Adenocarcinoma | Male | 52 | E19del | 4.17% | 1.5 | 80% |
| P302 | Adenocarcinoma | Female | 73 | L858R | 0.00% | 4.4 | ＜1% |
| P303 | Adenocarcinoma | Female | 45 | WT | 16.67% | 2.2 | 2% |
| P304 | Adenocarcinoma | Female | 72 | L858R | 8.33% | 4.4 | ＜1% |
| P305 | Adenocarcinoma | Male | 79 | WT | 4.17% | 7.4 | ＜1% |
| P306 | Adenocarcinoma | Female | 53 | E19del | 8.33% | 0.7 | 40% |
| P307 | Adenocarcinoma | Female | 67 | L858R | 0.00% | 5.9 | ＜1% |
| P308 | Adenocarcinoma | Female | 68 | WT | 4.17% | 2.9 | ＜1% |
| P309 | Adenocarcinoma | Male | 50 | L858R | 16.67% | 6.6 | 99% |
| P310 | Adenocarcinoma | Female | 64 | L858R | 4.17% | 1.5 | ＜1% |
| P311 | Adenocarcinoma | Female | 72 | L858R | 4.17% | 1.5 | 98% |
| P312 | Adenocarcinoma | Female | 80 | E19del | 8.33% | 8.1 | 10% |
| P313 | Adenocarcinoma | Male | 65 | WT | 4.17% | 1.5 | ＜1% |
| P314 | Squamous cell carcinoma | Male | 73 | other | 8.33% | 4.4 | ＜1% |
| P315 | Adenocarcinoma | Male | 64 | other | 4.17% | 5.1 | ＜1% |
| P316 | Adenocarcinoma | Male | 71 | WT | 4.17% | 13.2 | ＜1% |
| P317 | Adenocarcinoma | Male | 66 | WT | 4.17% | 5.1 | ＜1% |
| P318 | Adenocarcinoma | Male | 61 | L858R | 16.67% | 2.9 | ＜1% |
| P319 | Adenocarcinoma | Female | 60 | L858R | 4.17% | 2.9 | 15% |
| P320 | Adenocarcinoma | Female | 65 | E19del | 0.00% | 0.7 | ＜1% |
| P321 | Adenocarcinoma | Male | 64 | L858R | 0.00% | 0.7 | ＜1% |
| P322 | Adenocarcinoma | Male | 51 | WT | 4.17% | 2.2 | ＜1% |
| P323 | Adenocarcinoma | Male | 59 | L858R | 4.17% | 3.7 | 70% |
| P324 | Adenocarcinoma | Female | 63 | E19del | 0.00% | 1.5 | ＜1% |
| P325 | Squamous cell carcinoma | Male | 74 | WT | 8.33% | 5.9 | ＜1% |
| P326 | Adenocarcinoma | Male | 57 | L858R | 12.50% | 0.7 | 65% |
| P327 | Adenocarcinoma | Male | 66 | WT | 4.17% | 6.6 | 95% |
| P328 | Adenocarcinoma | Female | 54 | L858R | 4.17% | 0.7 | ＜1% |
| P329 | Squamous cell carcinoma | Male | 71 | WT | 4.17% | 9.6 | 80% |
| P330 | Squamous cell carcinoma | Male | 77 | WT | 4.17% | 6.6 | 80% |
| P331 | Adenocarcinoma | Female | 64 | L858R | 4.17% | 2.9 | 2% |
| P332 | Adenocarcinoma | Female | 67 | E19del | 4.17% | 1.5 | ＜1% |
| P333 | Adenocarcinoma | Male | 62 | E19del | 4.17% | 2.9 | ＜1% |
| P334 | Adenocarcinoma | Male | 40 | WT | 8.33% | 0.7 | ＜1% |
| P335 | Adenocarcinoma | Female | 69 | L858R | 0.00% | 2.9 | ＜1% |
| P336 | Adenocarcinoma | Female | 75 | WT | 0.00% | 2.9 | 65% |
| P337 | Adenocarcinoma | Male | 78 | WT | 0.00% | 11.8 | 99% |
| P338 | Adenocarcinoma | Female | 74 | L858R | 0.00% | 4.4 | ＜1% |
| P339 | Adenocarcinoma | Female | 78 | L858R | 0.00% | 2.2 | ＜1% |

**Table S2.** The gene list of AllNGS-Panel 639^TM^

| **Gene name** | | | | | | | | | | | | | | |
| --- | --- | --- | --- | --- | --- | --- | --- | --- | --- | --- | --- | --- | --- | --- |
| *ABCA6* | *ABCB1* | *[ABCC11](https://www.snpedia.com/index.php/ABCC11)* | *ABCC2* | *ABCC4* | *ABCF1* | *ABCG2* | *ABL1* | *ABL2* | *ACTR3B* | *ACVR1B* | *ADAMTS10* | *ADNP* | *AGAP9* | *AHNAK* |
| *AKAP7* | *AKR1C2* | *AKT1* | *AKT2* | *AKT3* | *ALK* | *AMER1* | *ANK2* | *ANKRD36* | *ANO10* | *APC* | *AR* | *ARAF* | *ARFRP1* | *ARHGAP5* |
| *ARID1A* | *ARID1B* | *ARID2* | *ART5* | *ASPM* | *ASXL1* | *ATM* | *ATR* | *ATRX* | *AURKA* | *AURKB* | *AXIN1* | *AXL* | *BAP1* | *BARD1* |
| *BAX* | *BCL2* | *BCL2L1* | *BCL2L11* | *BCL2L2* | *BCL6* | *BCL6B* | *BCOR* | *BCORL1* | *BCR* | *BEND5* | *BLM* | *BMPR2* | *BRAF* | *BRCA1* |
| *BRCA2* | *BRD3* | *BRD4* | *BRIP1* | *BTG1* | *BTK* | *C11orf30* | *C1orf144* | *C21orf58* | *C22orf31* | *C8orf34* | *CARD11* | *CASP5* | *CBFB* | *CBL* |
| *CBR3* | *CBWD6* | *CCDC144NL* | *CCKBR* | *CCND1* | *CCND2* | *CCND3* | *CCNE1* | *CCR5* | *CD274* | *CD3G* | *CD79A* | *CD79B* | *CDA* | *CDC27* |
| *CDC42EP1* | *CDC7* | *CDC73* | *CDCP2* | *CDH1* | *CDH5* | *CDK12* | *CDK4* | *CDK6* | *CDK8* | *CDKN1A* | *CDKN1B* | *CDKN2A* | *CDKN2B* | *CDKN2C* |
| *CEBPA* | *CENPH* | *CEP162* | *CEP164* | *CHD2* | *CHD4* | *CHEK1* | *CHEK2* | *CIC* | *CLASP1* | *CLDN16* | *CLIP1* | *CLOCK* | *CNDP1* | *COBLL1* |
| *COL5A3* | *CREBBP* | *CRKL* | *CRLF2* | *CROCC* | *CSF1R* | *CSMD3* | *CTCF* | *CTNNA1* | *CTNNB1* | *CUL3* | *CUZD1* | *CYB5R4* | *CYLD* | *CYP19A1* |
| *CYP1B1* | *CYP21A2* | *CYP2C19* | *CYP2C8* | *CYP2D6* | *CYP3A4* | *CYP3A5* | *CYP4B1* | *DAXX* | *DCP1B* | *DDHD1* | *DDR2* | *DDX11* | *DDX23* | *DEFB126* |
| *DHFR* | *DHX8* | *DICER1* | *DIEXF* | *DLEC1* | *DNMT3A* | *DOT1L* | *DPYD* | *DYNC2H1* | *EBPL* | *EGFR* | *EHBP1* | *ELFN1* | *EP300* | *EPCAM* |
| *EPHA3* | *EPHA5* | *EPHA7* | *EPHB1* | *ERBB2* | *ERBB3* | *ERBB4* | *ERCC1* | *ERCC2* | *ERG* | *ERRFI1* | *ESR1* | *ESR2* | *ESRRA* | *EZH2* |
| *F2RL2* | *FAM174B* | *FAM186A* | *FAM46C* | *FAM71E2* | *FANCA* | *FANCC* | *FANCD2* | *FANCE* | *FANCF* | *FANCG* | *FANCL* | *FAS* | *FAT1* | *FBXW7* |
| *FCAMR* | *FCGBP* | *FCGR3A* | *FCRLA* | *FGF10* | *FGF14* | *FGF19* | *FGF23* | *FGF3* | *FGF4* | *FGF6* | *FGFR1* | *FGFR2* | *FGFR3* | *FGFR4* |
| *FH* | *FKBP9* | *FLCN* | *FLT1* | *FLT3* | *FLT4* | *FMN2* | *FOPNL* | *FOXL2* | *FOXP1* | *FRS2* | *FUBP1* | *GABRA6* | *GATA1* | *GATA2* |
| *GATA3* | *GATA4* | *GATA6* | *GCNT2* | *GGH* | *GGT1* | *GID4* | *GLI1* | *GLTSCR1* | *GNA11* | *GNA13* | *GNAQ* | *GNAS* | *GNLY* | *GOLGA6L4* |
| *GOLGA6L6* | *GOT1L1* | *GPR124* | *GRIK2* | *GRIN2A* | *GRM3* | *GSK3B* | *GSTM5* | *[GSTO1](https://www.snpedia.com/index.php/Special:FormEdit/Gene/GSTO1)* | *GSTP1* | *H3F3A* | *HAVCR1* | *HGF* | *HIF1A* | *HIST1H3B* |
| *HLA* | *HMGXB4* | *HNF1A* | *HNRNPL* | *HRAS* | *HSD3B1* | *HSP90AA1* | *HSPA8* | *[HTR1E](https://www.snpedia.com/index.php/HTR1E)* | *IDH1* | *IDH2* | *IFI27* | *IFITM3* | *IGF1R* | *IGF2* |
| *IKBKE* | *IKZF1* | *IL7R* | *INHBA* | *INPP4B* | *IRF2* | *IRF4* | *IRS2* | *ISX* | *ITPA* | *JAK1* | *JAK2* | *JAK3* | *JPH4* | *JUN* |
| *KANK3* | *KAT6A* | *KAT6B* | *KCNB2* | *KCNJ5* | *KDM5A* | *KDM5C* | *KDM6A* | *KDR* | *KEAP1* | *KEL* | *KIAA0355* | *KIAA1024* | *KIAA1211* | *KIF25* |
| *KIF6* | *KIT* | *KLF4* | *KLHL6* | *KMT2A* | *KMT2C* | *KMT2D* | *KPNA2* | *KRAS* | *KRT15* | *KRT4* | *KRTAP10* | *KRTAP4* | *KRTAP5* | *LCE1F* |
| *LCE4A* | *LGALS9B* | *LIG1* | *LIMCH1* | *LMAN1* | *LMO1* | *LOC105374608* | *LOC107986229* | *LOR* | *LRP1B* | *LRP2* | *LTA* | *LURAP1L* | *LYN* | *LZTR1* |
| *M6PR* | *MAGI2* | *MAN1B1* | *MAP2K1* | *MAP2K2* | *MAP2K4* | *MAP3K1* | *MCCC2* | *MCHR2* | *MCL1* | *MCMDC2* | *MDM2* | *MDM4* | *MED12* | *MED13* |
| *MEF2B* | *MEN1* | *MET* | *MITF* | *MLH1* | *MLLT3* | *MPL* | *MRE11A* | *MSH2* | *MSH3* | *MSH6* | *MTHFR* | *MTOR* | *MUC2* | *MUC4* |
| *MUC6* | *MUTYH* | *MVK* | *MYB* | *MYC* | *MYCL* | *MYCN* | *MYD88* | *MYL1* | *MYOM1* | *NBEA* | *NBN* | *NCOA3* | *NCOA6* | *NCOR2* |
| *NEFH* | *NF1* | *NF2* | *NFE2L2* | *NFKBIA* | *NFXL1* | *NIPBL* | *NKX2-1* | *NOTCH1* | *NOTCH2* | *NOTCH3* | *NPM1* | *NQO1* | *NR1H2* | *NRAS* |
| *NRP2* | *NSD1* | *NTRK1* | *NTRK2* | *NTRK3* | *NUP155* | *NUP93* | *OPRK1* | *OR11H4* | *OR2B11* | *OR52D1* | *OR5K4* | *OR6C76* | *OR8I2* | *ORAI1* |
| *PAK3* | *PALB2* | *PARK2* | *PAX5* | *PBRM1* | *PCDH12* | *PDCD1LG2* | *PDE11A* | *PDE7A* | *PDGFRA* | *PDGFRB* | *PDK1* | *PHGR1* | *PIK3C2B* | *PIK3C3* |
| *PIK3CA* | *PIK3CB* | *PIK3CG* | *PIK3R1* | *PIK3R2* | *PIP4K2A* | *PKD1L2* | *PLCG2* | *PMS2* | *POLD1* | *POLE* | *POLI* | *POTEC* | *PPP2R1A* | *PPP6C* |
| *PRDM1* | *PREX2* | *PRKAR1A* | *PRKCH* | *PRKCI* | *PRKDC* | *PRKRA* | *PROSER3* | *PRPF19* | *PRSS8* | *PRX* | *PTCH1* | *PTEN* | *PTGS2* | *PTPN11* |
| *QKI* | *RAC1* | *RAD50* | *RAD51* | *RAF1* | *RALY* | *RANBP2* | *RARA* | *RB1* | *RBM10* | *RBM27* | *RBM5* | *RET* | *RETNLB* | *RFX3* |
| *RGPD3* | *RGS12* | *RHPN2* | *RIC8A* | *RICTOR* | *RIN3* | *RNF145* | *RNF213* | *RNF43* | *ROCK1* | *ROS1* | *RP1L1* | *RPL8* | *RPS12* | *RPS9* |
| *RPTN* | *RPTOR* | *RRM1* | *RSBN1L* | *RUNX1* | *RUNX1T1* | *SCAI* | *SCYL2* | *SDHA* | *SDHB* | *SDHC* | *SDHD* | *SEC31A* | *SELE* | *SELPLG* |
| *[SEMA3C](https://www.snpedia.com/index.php/Special:FormEdit/Gene/SEMA3C)* | *SEMA5B* | *SEPP1* | *SERPINA10* | *SETBP1* | *SETD2* | *SF3B1* | *SH3GL1* | *SI* | *SIK2* | *SKIDA1* | *SLAMF1* | *SLC11A2* | *SLC19A1* | *SLC22A2* |
| *SLC28A3* | *SLC29A1* | *SLC2A5* | *SLC35F5* | *SLC35G2* | *SLC36A2* | *SLC3A2* | *SLC6A18* | *SLCO1B3* | *SLIT2* | *SLK* | *SLX4* | *SMAD2* | *SMAD3* | *SMAD4* |
| *SMARCA4* | *SMARCB1* | *SMO* | *SNCAIP* | *SNX13* | *SOCS1* | *SOD2* | *SOX10* | *SOX11* | *SOX2* | *SOX9* | *SPATA3* | *SPEN* | *SPOP* | *SPTA1* |
| *SRBD1* | *SRC* | *SRD5A2* | *SRGAP3* | *SRPR* | *SRSF2* | *SSTR4* | *ST18* | *STAG2* | *STAT3* | *STAT4* | *STK11* | *STMN1* | *SUFU* | *SULT1A1* |
| *SVIL* | *SYK* | *TAF1* | *TAF1B* | *TBC1D23* | *TBK1* | *TBX3* | *TCERG1* | *TEAD2* | *TERC* | *TERT* | *TET2* | *TFAM* | *TGFBR2* | *THAP2* |
| *THAP3* | *THAP5* | *TMBIM4* | *TMEM106B* | *TMEM37* | *TMEM60* | *TMEM97* | *TNFAIP3* | *TNFAIP6* | *TNFRSF14* | *TOMM70* | *TOP1* | *TOP2A* | *TP53* | *TPMT* |
| *TRIM48* | *TRIM51* | *TRMT10C* | *TRRAP* | *TSC1* | *TSC2* | *TSHR* | *TTK* | *TTLL10* | *TVP23A* | *TXNDC2* | *TYMS* | *U2AF1* | *UBA7* | *UBE3C* |
| *UBE4A* | *UBR5* | *UGT1A1* | *UHRF1* | *ULK4* | *UMPS* | *UPF3A* | *USP35* | *USP36* | *VEGFA* | *VEZT* | *VHL* | *VIT* | *WDR37* | *WDR66* |
| *WDTC1* | *WISP3* | *WT1* | *XPC* | *XPO1* | *XRCC1* | *XRCC2* | *XYLT2* | *ZBTB2* | *ZFP37* | *ZFR2* | *ZNF217* | *ZNF365* | *ZNF429* | *ZNF462* |
| *ZNF479* | *ZNF516* | *ZNF527* | *ZNF605* | *ZNF703* | *ZNF717* | *ZNF776* | *ZNF814* | *ZNF844* |  |  |  |  |  |  |

**Table S3. Association between TMB status and clinical features**.

| **Characteristics** | **TMB status** | | **P value** |
| --- | --- | --- | --- |
|  | **TMB-H**  **(N=110)** | **TMB-L**  **(N=229)** |  |
| **Pathological type.** |  |  | <0.001 |
| Adenocarcinoma. | 77 ( 70.0%) | 218 ( 95.2%) |  |
| Squamous cell carcinoma | 33 ( 30.0%) | 11 ( 4.8%) |  |
| **Gender** |  |  | <0.001 |
| Female | 31 ( 28.2%) | 138 ( 60.3%) |  |
| Male | 79 ( 71.8%) | 91 ( 39.7%) |  |
| **Age at diagnosis in years** |  |  | <0.001 |
| 60- (< 60) | 26 ( 23.6%) | 105 ( 45.9%) |  |
| 60+ (≥ 60) | 84 ( 76.4%) | 124 ( 54.1%) |  |
| **EGFR status** |  |  | <0.001 |
| E19del | 11 ( 10.0%) | 55 ( 24.0%) |  |
| L858R | 18 ( 16.4%) | 84 ( 36.7%) |  |
| other | 13 ( 11.8%) | 15 ( 6.6%) |  |
| Wild type | 68 ( 61.8%) | 75 ( 32.8%) |  |
| **PD-L1 status** |  |  | 0.003 |
| High ( > 50%) | 36 ( 32.7%) | 38 ( 16.6%) |  |
| Medaium (1% ~50%) | 24 ( 21.8%) | 55 ( 24.0%) |  |
| Negative ( < 1%) | 50 ( 45.5%) | 136 ( 59.4%) |  |
| **MSI status** |  |  | 0.514 |
| MSI-H | 2 ( 1.8%) | 1 ( 0.4%) |  |
| MSS | 108 ( 98.2%) | 228 ( 99.6%) |  |

**Table S4** Differential analysis of mutations in signaling pathways related to EGFR-sensitive mutations or high PD-L1 expression.

| **Pathway** | **Without E19del /L858R mutation** | | **With E19del /L858R mutation** | | **P-value** |
| --- | --- | --- | --- | --- | --- |
|  | **PD-L1 < 50%**  **(N=123)** | **PD-L1 ≥ 50%**  **(N=48)** | **PD-L1 < 50%**  **(N=142)** | **PD-L1 ≥ 50%**  **(N=26)** |  |
| **Apoptosis (mean (SD))** | 0.00 (0.00) | 0.00 (0.00) | 0.00 (0.00) | 0.00 (0.00) | NaN |
| **Cell cycle (mean (SD))** | 1.42 (3.99) | 1.82 (4.46) | 0.62 (2.72) | 0.96 (3.40) | 0.129 |
| **Chromatin histone modifiers (mean (SD))** | 1.36 (3.19) | 2.08 (3.93) | 0.66 (2.15) | 0.51 (1.81) | 0.011 |
| **Chromatin other (mean (SD))** | 0.29 (1.42) | 0.00 (0.00) | 0.10 (0.84) | 0.00 (0.00) | 0.233 |
| **Chromatin SWI/SNF complex (mean (SD))** | 1.32 (4.18) | 1.04 (3.49) | 0.53 (2.52) | 0.96 (3.40) | 0.304 |
| **Epigenetics DNA modifiers (mean (SD))** | 0.81 (9.02) | 2.08 (14.43) | 0.00 (0.00) | 0.00 (0.00) | 0.406 |
| **Genome integrity (mean (SD))** | 4.88 (5.36) | 5.80 (5.64) | 3.32 (4.22) | 3.85 (4.16) | 0.007 |
| **Histone modification (mean (SD))** | 0.81 (5.16) | 4.17 (11.14) | 0.23 (2.80) | 1.28 (6.54) | 0.001 |
| **Immune signaling (mean (SD))** | 0.08 (0.90) | 0.21 (1.44) | 0.00 (0.00) | 0.00 (0.00) | 0.406 |
| **MAPK signaling (mean (SD))** | 2.89 (4.89) | 3.94 (6.68) | 0.23 (1.60) | 0.00 (0.00) | <0.001 |
| **Metabolism (mean (SD))** | 0.00 (0.00) | 0.00 (0.00) | 0.00 (0.00) | 0.00 (0.00) | NaN |
| **NFKB signaling (mean (SD))** | 0.41 (4.51) | 0.00 (0.00) | 0.00 (0.00) | 0.00 (0.00) | 0.626 |
| **NOTCH signaling (mean (SD))** | 2.44 (15.49) | 0.00 (0.00) | 0.00 (0.00) | 0.00 (0.00) | 0.151 |
| **Other (mean (SD))** | 0.30 (1.13) | 0.47 (1.40) | 0.13 (0.75) | 0.35 (1.24) | 0.206 |
| **Other signaling (mean (SD))** | 0.75 (1.60) | 1.12 (1.97) | 0.20 (1.02) | 0.14 (0.70) | <0.001 |
| **PI3K signaling (mean (SD))** | 1.26 (3.82) | 2.08 (4.95) | 1.33 (3.62) | 4.27 (5.51) | 0.004 |
| **Protein homeostasis/ubiquitination (mean (SD))** | 0.16 (1.03) | 0.56 (1.86) | 0.14 (0.96) | 0.00 (0.00) | 0.107 |
| **RNA abundance (mean (SD))** | 0.38 (1.55) | 1.25 (2.63) | 0.47 (1.71) | 0.51 (1.81) | 0.039 |
| **RTK signaling (mean (SD))** | 2.69 (4.53) | 2.60 (4.24) | 6.29 (0.91) | 6.25 (0.00) | <0.001 |
| **Splicing (mean (SD))** | 0.68 (3.30) | 0.00 (0.00) | 0.00 (0.00) | 0.00 (0.00) | 0.03 |
| **TGFB signaling (mean (SD))** | 0.70 (3.09) | 0.60 (2.88) | 0.20 (1.69) | 0.00 (0.00) | 0.284 |
| **TOR signaling (mean (SD))** | 2.98 (10.46) | 0.00 (0.00) | 0.70 (4.81) | 0.00 (0.00) | 0.016 |
| **Transcription factor (mean (SD))** | 0.27 (0.86) | 0.32 (0.86) | 0.22 (0.72) | 0.10 (0.50) | 0.639 |
| **Wnt/B catenin signaling (mean (SD))** | 1.32 (4.47) | 0.78 (3.06) | 0.88 (3.21) | 1.44 (4.07) | 0.695 |

**Table S5** Differential analysis of mutations in signaling pathways related to EGFR-sensitive mutations or TMB-H.

| **Pathway** | **Without E19del /L858R mutation** | | **With E19del /L858R mutation** | | **P-value** |
| --- | --- | --- | --- | --- | --- |
|  | **TMB < 4.4 muts/Mb**  **(N=90)** | **TMB ≥ 4.4 muts/Mb**  **(N=81)** | **TMB < 4.4 muts/Mb**  **(N=139)** | **TMB ≥ 4.4 muts/Mb**  **(N=29)** |  |
| **Apoptosis (mean (SD))** | 0.00 (0.00) | 0.00 (0.00) | 0.00 (0.00) | 0.00 (0.00) | NaN |
| **Cell cycle (mean (SD))** | 0.69 (2.88) | 2.47 (5.01) | 0.45 (2.34) | 1.72 (4.39) | <0.001 |
| **Chromatin histone modifiers (mean (SD))** | 0.67 (2.25) | 2.55 (4.16) | 0.53 (1.81) | 1.15 (3.12) | <0.001 |
| **Chromatin other (mean (SD))** | 0.16 (1.06) | 0.26 (1.36) | 0.10 (0.85) | 0.00 (0.00) | 0.582 |
| **Chromatin SWI/SNF complex (mean (SD))** | 0.69 (2.88) | 1.85 (4.89) | 0.36 (2.10) | 1.72 (4.39) | 0.008 |
| **Epigenetics DNA modifiers (mean (SD))** | 0.00 (0.00) | 2.47 (15.61) | 0.00 (0.00) | 0.00 (0.00) | 0.094 |
| **Genome integrity (mean (SD))** | 2.94 (3.99) | 7.58 (5.80) | 2.88 (3.82) | 5.91 (5.08) | <0.001 |
| **Histone modification (mean (SD))** | 1.85 (7.68) | 1.65 (7.27) | 0.24 (2.83) | 1.15 (6.19) | 0.166 |
| **Immune signaling (mean (SD))** | 0.00 (0.00) | 0.25 (1.56) | 0.00 (0.00) | 0.00 (0.00) | 0.094 |
| **MAPK signaling (mean (SD))** | 1.98 (4.27) | 4.53 (6.28) | 0.16 (1.33) | 0.38 (2.06) | <0.001 |
| **Metabolism (mean (SD))** | 0.00 (0.00) | 0.00 (0.00) | 0.00 (0.00) | 0.00 (0.00) | NaN |
| **NFKB signaling (mean (SD))** | 0.00 (0.00) | 0.62 (5.56) | 0.00 (0.00) | 0.00 (0.00) | 0.365 |
| **NOTCH signaling (mean (SD))** | 1.11 (10.54) | 2.47 (15.61) | 0.00 (0.00) | 0.00 (0.00) | 0.278 |
| **Other (mean (SD))** | 0.10 (0.67) | 0.62 (1.57) | 0.10 (0.66) | 0.47 (1.41) | 0.001 |
| **Other signaling (mean (SD))** | 0.36 (1.08) | 1.41 (2.09) | 0.08 (0.68) | 0.74 (1.75) | <0.001 |
| **PI3K signaling (mean (SD))** | 0.49 (2.30) | 2.61 (5.35) | 1.44 (3.74) | 3.45 (5.23) | 0.001 |
| **Protein homeostasis/ubiquitination (mean (SD))** | 0.15 (0.99) | 0.41 (1.61) | 0.14 (0.97) | 0.00 (0.00) | 0.233 |
| **RNA abundance (mean (SD))** | 0.30 (1.38) | 0.99 (2.38) | 0.48 (1.73) | 0.46 (1.72) | 0.086 |
| **RTK signaling (mean (SD))** | 2.08 (3.63) | 3.32 (5.14) | 6.25 (0.75) | 6.47 (1.16) | <0.001 |
| **Splicing (mean (SD))** | 0.37 (2.47) | 0.62 (3.17) | 0.00 (0.00) | 0.00 (0.00) | 0.132 |
| **TGFB signaling (mean (SD))** | 0.32 (2.12) | 1.06 (3.76) | 0.10 (1.21) | 0.49 (2.65) | 0.041 |
| **TOR signaling (mean (SD))** | 1.48 (6.91) | 2.88 (10.80) | 0.72 (4.86) | 0.00 (0.00) | 0.116 |
| **Transcription factor (mean (SD))** | 0.14 (0.59) | 0.44 (1.06) | 0.15 (0.60) | 0.44 (0.99) | 0.012 |
| **Wnt/B catenin signaling (mean (SD))** | 0.97 (3.85) | 1.39 (4.42) | 0.81 (3.09) | 1.72 (4.39) | 0.535 |
